# Fully Flexible Docking of Medium Sized Ligand Libraries with RosettaLigand

**DOI:** 10.1371/journal.pone.0132508

**Published:** 2015-07-24

**Authors:** Samuel DeLuca, Karen Khar, Jens Meiler

**Affiliations:** 1 Department of Chemistry, Vanderbilt University, Nashville, TN, United States of America; 2 Center for Computational Biology, University of Kansas, Lawrence, KS, United States of America; University of Michigan, UNITED STATES

## Abstract

RosettaLigand has been successfully used to predict binding poses in protein-small molecule complexes. However, the RosettaLigand docking protocol is comparatively slow in identifying an initial starting pose for the small molecule (ligand) making it unfeasible for use in virtual High Throughput Screening (vHTS). To overcome this limitation, we developed a new sampling approach for placing the ligand in the protein binding site during the initial ‘low-resolution’ docking step. It combines the translational and rotational adjustments to the ligand pose in a single transformation step. The new algorithm is both more accurate and more time-efficient. The docking success rate is improved by 10–15% in a benchmark set of 43 protein/ligand complexes, reducing the number of models that typically need to be generated from 1000 to 150. The average time to generate a model is reduced from 50 seconds to 10 seconds. As a result we observe an effective 30-fold speed increase, making RosettaLigand appropriate for docking medium sized ligand libraries. We demonstrate that this improved initial placement of the ligand is critical for successful prediction of an accurate binding position in the ‘high-resolution’ full atom refinement step.

## Introduction

Computational ligand docking has been a historically successful method for predicting the binding position of small molecules to a protein. Beginning with PJ Goodford’s work in computational drug design [1], many methods have been developed to predict interactions between proteins and small molecules. Early tools focused primarily on rigid body shape complementarity between a small molecule ligand and a binding pocket in an experimental protein structure. However, changes observed in protein conformation upon the binding of a small molecule [2] suggested that modeling of protein and ligand flexibility were important to correctly model protein/ligand interactions.

Over the past several decades, numerous tools have been developed to better address the ligand docking problem. DOCK [3], FlexX [4], AutoDock [5], and Glide [6] are currently among the most popular tools. These tools utilize a wide range of protein representations, sampling algorithms and scoring functions to accurately predict protein/ligand binding geometry. Approximations in scoring and sampling must be made in order for simulations to be completed in a reasonable time. To accomplish this, most ligand docking tools operate in multiple stages of reducing the conformational search space, increasing sampling density, and increasing complexity of the scoring function.

### Summary of popularly used docking algorithms

Ligand docking methods differ in their means of increasing score function complexity while reducing search space. For example, the DOCK algorithm creates a “negative space” model of the binding site by placing spheres inside the solvent accessible area of the binding site. This model is used to guide docking of the ligand, while an Assisted Model Building with Energy Refinement (AMBER) based molecular mechanics force field is used to score the resulting binding positions [7].

FlexX, on the other hand, represents the protein as “interaction centers” consisting of surfaces surrounding common ligand interaction groups (hydrogen bond donors and acceptors, metals, aromatic rings, etc.). The ligand is then broken into fragments, and atoms in the fragments are matched to the interaction centers to provide an ensemble of potential initial placements [8].

AutoDock represents the protein using a Cartesian scoring grid populated with information from an empirically derived energy function. A Lamarckian Genetic Algorithm (LGA) in combination with simulated annealing is then used to optimize both the ligand conformation and position [5].

Glide uses a grid based representation of the protein binding site. A rapid exhaustive search is performed to find generally favorable areas for ligand placement. A size filter is then used to exclude areas without sufficient space for ligand placement. Finally, Monte Carlo Minimization (MCM) of the binding position using the grid based scoring function is performed. The scoring girds themselves are generated using a scoring function derived from ChemScore [6].

### Performance of ligand docking tools is inconsistent

Despite the differences in scoring and sampling algorithm implementations across the different ligand docking tools, a blind study of ligand docking performance conducted by Davis et al. [9] suggested that while certain methods of docking perform better than others for a given protein target, in aggregate the commonly used systems have a similar performance. While the overall performance is similar, the performance of varies greatly from one protein/ligand complex to the next. While some protein systems appear to be generally successful (Chk1 kinase) or generally difficult (Hepatitis C RNA Polymerase), for most protein/ligand complexes results vary depending on the ligand docking tool used. The difficulty of predicting whether a given docking tool will perform well on a given protein/ligand complex has been previously discussed [10,11]. There is a clear need for consistently reliable protein/ligand docking tools.

### Limitations of RosettaLigand low resolution docking

The work presented in this manuscript consists of a set of improvements to the RosettaLigand docking algorithm [9,12–14]. RosettaLigand uses a two stage docking process consisting of an initial placement stage followed by a refinement stage. The goal of the initial placement stage is to place the ligand in a non-clashing position at random. The initial placement algorithm consists of three steps, which are described in Davis et al. [15] The algorithm uses a scoring grid to identify non-clashing regions of the protein. Two sets of binary scoring grids are combined: “Repulsive” shells with a radius of 2.25 Å around each backbone heavy atom of the protein and “attractive” shells between 2.25 and 4.75 Å around each heavy atom.

The first step of the initial placement algorithm (“Translate”) consists of up to 50 random translations within 5.0 Å of the starting position. After each translation step, the heavy atom closest to the geometric center of the ligand, termed the “neighbor atom”, is scored using the binary scoring grid. If the score is -1 or 0 (attractive or neutral) the move is accepted and the translation step terminates. The aim of the Translate step is to place the ligand in a region of the binding site that lacks a severe clash with the protein.

The second step in the initial placement algorithm is the “Rotate” step. The Rotate step consists of up to 500 random rotations of variable magnitude up to complete reorientation (360°) from the starting orientation. The Rotate step accumulates a set of diverse non-clashing ligand orientations, and then selects one of these orientations at random for further refinement. The size of the set of diverse orientations is either 5 or 5 times the number of rotatable bonds in the ligand, whichever is larger. The ligand is randomly reoriented and then accepted into the set of diverse orientations upon the following conditions: No atoms are located in repulsive squares, 85% of the atoms are located on attractive squares, and the Root Mean Square Deviation (RMSD) of the new orientation with respect to all previously accepted conformations is greater than 0.65 x square root of the number of heavy atoms. After either 500 placements have been created or the maximum set size has been achieved, a random orientation from the set is selected, and the Rotate step terminates.

The third and final step is the “Slide Together” step. During the prior two steps, it is possible for the ligand to be placed in a region where it fails to contact the protein. In this case the apparent interaction energy at the beginning of the refinement stage would be 0, reducing the efficiency and sometimes causing failure in the following Monte Carlo refinement stage. To avoid this situation, the Slide Together step moves the ligand towards the center of mass of the protein as long as the full atom repulsive score remains zero. We will refer to this algorithm as ‘TransRot’. Following this initial placement, a refinement stage is carried out in which small perturbations of the ligand and repacking of the protein side-chains are performed using MCM. Finally, position of all atoms in the protein/ligand interface are optimized using gradient minimization on the RosettaLigand energy function.

### Limitations of the low resolution placement in RosettaLigand

**Fig 1:**
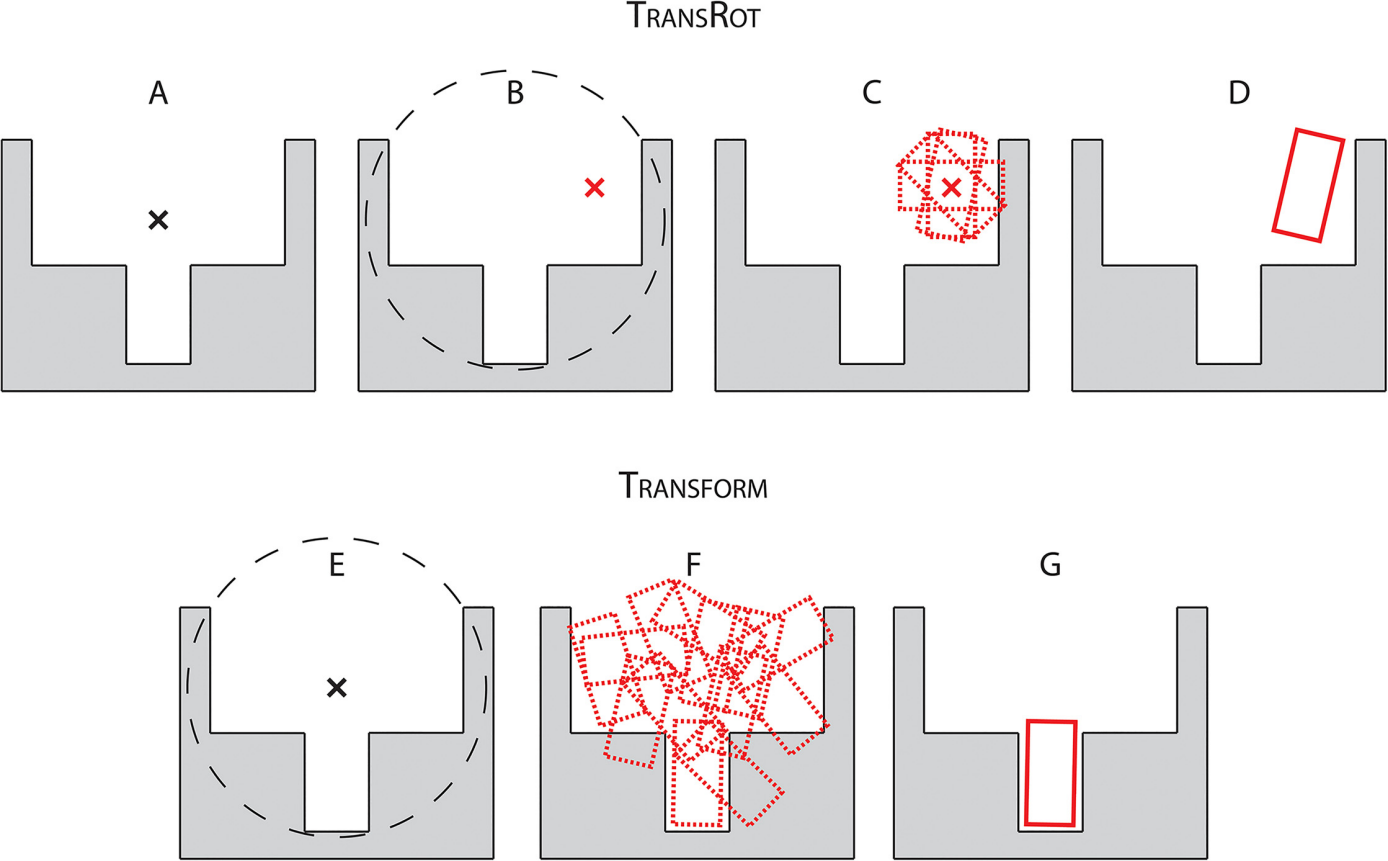
A schematic indicating the hypothetical mechanism by which the Transform algorithm exhibits improved performance compared to the TransRot algorithm. A) When the TransRot algorithm is used, a Cartesian starting coordinate is specified as the starting position for the ligand. B) This starting point is then translated to a random location which does not overlap with the protein backbone. C) The ligand is centered at the new random location within a user specified starting radius, and a set of diverse, minimally- clashing rotational binding positions are selected. D) A single random binding pose is selected for refinement. E) When the Transform algorithm is used, the starting Cartesian coordinate is specified as the starting position for the ligand. F) The simultaneous translations and rotations within a user specified radius is sampled using a MCM algorithm. G) The best scoring model is selected from step (F) for refinement.

We hypothesized that the independent translation and rotation stages complicate sampling of all favorable initial placements, particularly if the ligand is not globular. For example, a rod-shaped ligand would easily enter a rod-shaped pocket but only if it is brought into the correct orientation first. A ligand with a bent shape might require reorientation while entering the binding pocket in order to avoid clashes. Therefore, RosettaLigand will miss out on favorable initial placements for certain ligands and spend unnecessary time performing refinement and minimization moves. Fig 1 schematically illustrates this hypothesis. The Translate step described above only takes into account the geometric center of the ligand. As a result, a ligand that needs to enter a narrow binding pocket is likely to be initially translated in unfavorable relative orientations with respect to the pocket entrance (Fig 1B). Once the ligand has been translated into an undesirable locations the rotational sampling (Fig 1C) has no way of arriving at a high quality binding pose. The unfavorable translation can only be corrected by beginning with a new binding position (Fig 1D). The result of this inefficiency in sampling would be an increased failure rate as some ligands are never placed in favorable starting positions. For other ligands, an increased runtime is observed as the number of ligand binding positions which must be generated to reliably produce a high quality binding position is increased. Lemmon et al. [16] determined that as many as 1000 models may be necessary to produce at least one high quality binding position in a challenging docking case. Given this, improving the efficiency of sampling ligand poses in the protein binding site has the potential to drastically reduce the computational cost of RosettaLigand, allowing for a larger number of predictions to be made given a fixed amount of computing resources. The new Transform algorithm presented in this manuscript samples both translation and rotation simultaneously (Fig 1F) and increases the likelihood of arriving at a reasonable binding conformation relative to the separate translation and rotation steps of the previously published TransRot algorithm.

## Results and Discussion

The improved initial placement algorithm, here referred to as the Transform algorithm, has two independent components: A modular grid based scoring function, and a MCM based sampling algorithm. The software is implemented so as to allow for the rapid development of new score terms and sampling methodologies and the easy integration of these methods into the existing RosettaLigand modeling pipeline.

### Scoring grids and manager

Scoring of ligand binding positions in the new Transform algorithm is handled using scoring grids that are controlled by a scoring manager (S1 Fig). Each scoring grid is responsible for computing a single term in the grid based energy function. In this study, a single scoring grid, identical to that used by the TransRot algorithm, was used for scoring. The scoring manager consists of a three-dimensional tensor of floating point values representing Cartesian space, functions to populate the tensor, and functions to score ligands positioned in it. The scoring manager is responsible for keeping the scoring grid up to date with respect to the protein binding position, and for evaluating the score of the ligand based on the grid. For this study, the tensor is a 1000 Å^3^ cube, with a spacing of 0.25 Å between grid points was used. While the size and density of the grid were not rigorously optimized in this study, we derived the following guidelines for setting parameters: The size of the grid must be sufficiently large that if the ligand is translated as to the maximum allowed range, (5.0 Å in this study) every ligand atom will exist within the grid. RosettaLigand will reject any move that would result in ligand atoms placed outside the grid; hence an overly small grid will artificially constrain ligand sampling. On the other hand, the amount of memory necessary to store a scoring grid increases with the cube of the grid side length. The CPU can handle smaller scoring grids more efficiently; hence a scoring grid that is too large may result in a substantial decrease in algorithm speed. Similarly, the spacing between grid points must be small enough to capture the differences between nearby atoms, but not so small that the grid is too large to be efficiently handled. Overall, a scoring grid should be large enough to encompass the entire protein/ligand binding site, but no larger.

### Description of Monte Carlo Metropolis algorithm to place Ligand in Grid during RosettaLigand low resolution docking

**Fig 2:**
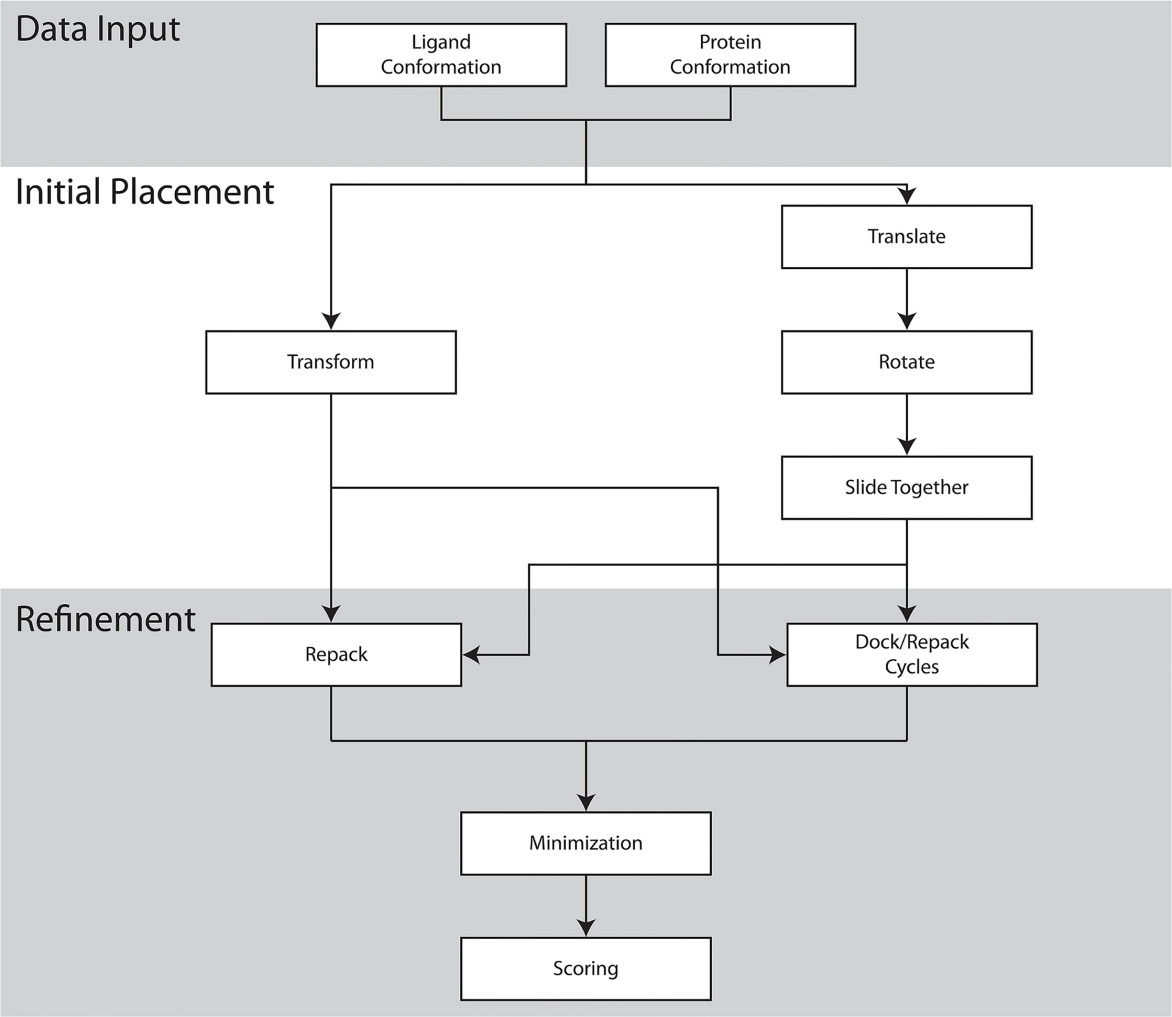
A schematic of the Transform (left) and TransRot (right) docking protocols described in this paper. Because the initial placement and refinement steps are independent, the two initial placement algorithms can be alternatively selected to produce a total of four ligand docking algorithms.

An MCM algorithm is used to determine the initial binding position for the ligand that will be used as the starting point for the second atomic-detail refinement stage. Fig 2 shows a flow chart of the overall steps in the RosettaLigand protocol. At each step in the sampling process the ligand is either randomly perturbed in the binding site, or the conformation of the ligand is changed. Ligand perturbation is performed as a combination of a random translation and rotation, and the conformation of the ligand is perturbed by selecting a random conformation from a library of pre-computed conformers. After the perturbation or conformation change, the ligand is scored using the grids described above, and the Metropolis criterion is applied to accept or reject the new binding position. After 500 cycles of sampling are performed, the best scoring ligand pose is kept. During this stage of sampling, only the scoring grids are used to provide scoring information, and the protein is kept rigid. By only using scoring grid information, it is possible to perform 500 cycles of sampling in roughly 1–3 seconds. This study compares different configurations of both the initial placement step and the refinement step, described below. Complete RosettaScripts eXtensible Markup Language (XML) files for each experiment can be found in in the supplemental materials (S1 Protocol Capture). As a baseline for performance comparisons we use the previously published TransRot initial placement algorithm, in which translation and rotation moves are performed separately using the binary scoring grids. The specific TransRot algorithm used here is originally described in Fleishman et al. [17], and is functionally identical to the process described in the Davis paper, though the user interface is different.

### Description of the atomic detail refinement algorithms tested for stage 2

We tested two refinement algorithms: Monte-Carlo Metropolis (MCM) and ‘minimization’ (MIN). For both algorithms the full atom Rosetta energy function is used for scoring than the scoring grids. In MCM refinement, six steps of high resolution docking are performed. Steps 1, 3, and 5 consist of repacking followed by minimization, while steps 2, 4, and 6 consist of small perturbations of the ligand. In the repacking and minimization step, the side-chain positions are optimized using rotamers from the Dunbrack library [18], and the ligand is allowed to change conformation using a set of pre-computed conformers. Following repacking, a gradient based minimization is applied to minimize the energy of the side-chain and ligand atoms. In the perturbation step, the ligand is randomly perturbed within a range of 0.1 Å and rotated up to 20° per move. The best scoring binding position of the six moves is kept. After high resolution docking, a final minimization step is carried out in which the protein side-chain and backbone atoms in the binding site, as well as the ligand atoms, are minimized using a gradient algorithm.

Minimization (MIN) refinement is carried out similarly to MCM refinement, except that in only a single round of repacking is performed prior to the final gradient minimization. Because no ligand perturbation is performed during MIN refinement, the ligand position generated during the initial placement is critical to the final binding pose and score.

### Overview of the CSAR benchmarking dataset

To benchmark the performance of the new initial placement algorithm, a subset of 43 protein/ligand complexes from the docking benchmark derived from the Community Structure-Activity Resource (CSAR) [19] dataset was used (S1 Table) The ligands in the CSAR subset have binding affinities ranging from-log(K_d_) of 2.1 to 9.4, and molecular weights ranging from 81 to 488. Additionally, the range of rotatable bonds in the subset was 0–3, the range of hydrogen bond donors was 0–8, and the range of hydrogen bond acceptors was 1–10. This subset omits protein/ligand complexes with co-factors, metal ions, or water molecules that bridge ligand and protein. While Rosetta has been used in such cases [16], the inclusion of water molecules, co-factors, or metal ions increases the number of degrees of freedom in the docking simulation and would complicate interpretation of the results.

### Description of the three sets of input models used in the CSAR based benchmark

Because the new initial placement algorithm relies on a pre-computed scoring grid, the initial positions of the protein atoms have an impact on the quality of the generated binding positions. To assess the extent of this impact, three sets of input structures were used in docking: the experimental structures provided in the CSAR dataset, repacked structures in which the backbone was held fixed and the side-chains re-optimized without the ligand present, and relaxed structures in which both the side-chain and backbone atoms were minimized within Rosetta in absence of the small molecule. In the case of the experimental and repacked structures, only a single protein structure was used for docking. In the case of the relaxed structures, the ligand was docked into an ensemble of ten models.

### Twelve benchmark experiments were performed

Each experiment is a combination of one of three possible input protein structures described above (experimental, repacked, relaxed) and one of four docking protocols. A docking protocol consists of an initial placement algorithm (TransRot or Transform), and a refinement algorithm (MCM or MIN). Fig 2 is a schematic description of the overall docking process.

### The Transform algorithm decreases the amount of time required to make one model

**Fig 3:**
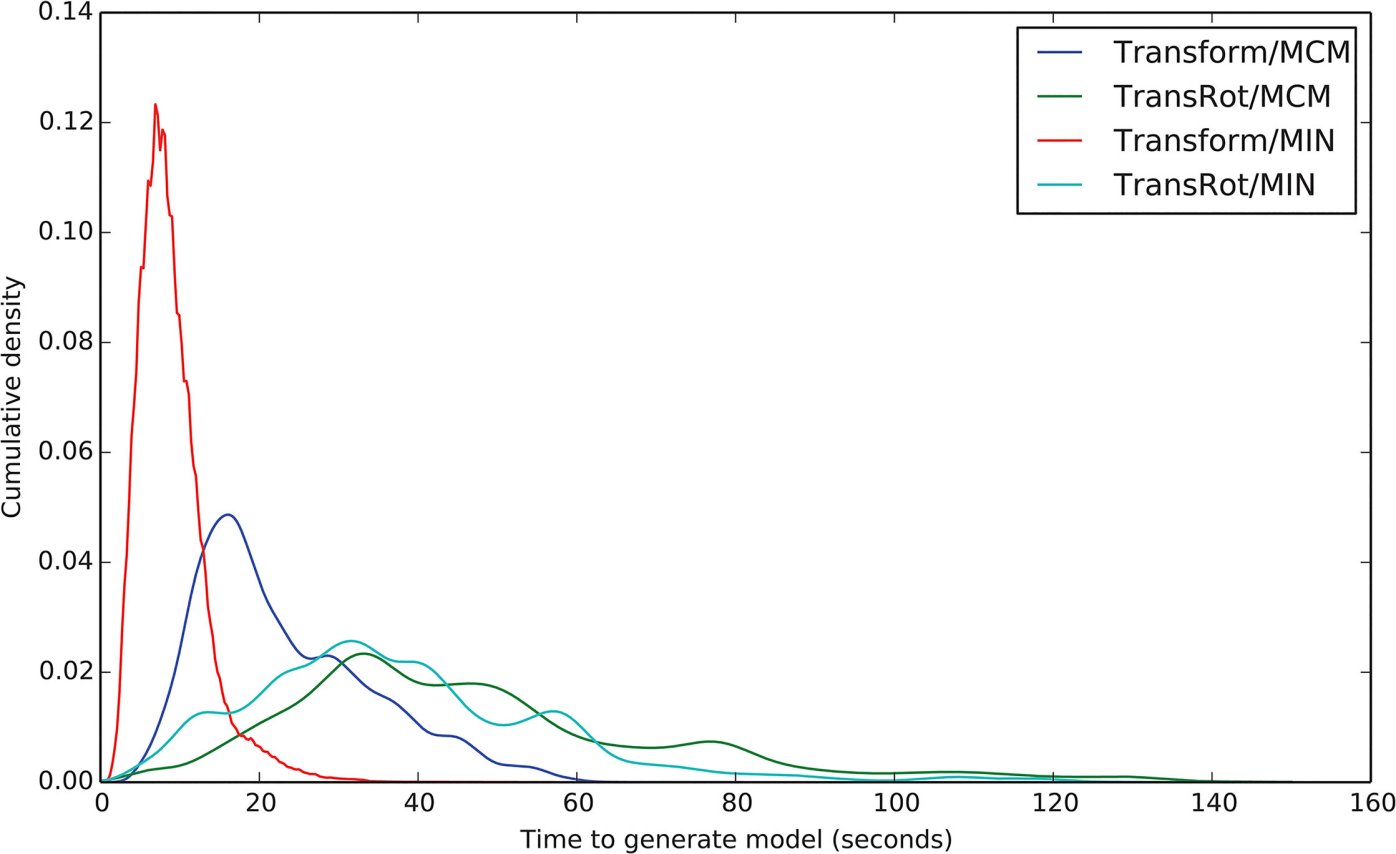
Kernel Density Estimate curves showing the time necessary to generate a single model using the four RosettaLigand protocols. TransRot/MCM is the protocol previously published by Davis et al. [15].

Fig 3 shows the time necessary to generate a single model with each of the four tested algorithms. The average time needed to generate a model using the previously published TransRot/MCM protocol is 49.4 seconds per model. Changing the Refinement protocol from MCM to MIN reduces the time per model to 33.3 seconds, and changing both the refinement protocol to MIN and the initial placement model from TransRot to Transform further reduces the time per model to 9.3 seconds. The per-model timing is not uniformly distributed, and varies based on the docking protocol used. The standard deviations of the time to generate models using the Transform algorithms are lower than those of the TransRot algorithms. Specifically, the time distribution for the generation of Transform/MCM models has a standard deviation of 10.5 seconds and the standard deviation of the distribution for Transform/MIN is 4.0 seconds. On the other hand, the timing distribution of the TransRot/MCM models has a standard deviation of 26.6 seconds, and the timing distribution of the TransRot/MIN models has a standard deviation of 21.0 seconds.

In addition to a narrower timing distribution, the choice of algorithm also affects the skewness of the distribution. Specifically, the timing distribution for models generated using Transform algorithm exhibit a lower skewness value and therefore a more normal distribution than models generated using the TransRot algorithm. Skewness values are 1.7 and 0.7 for the Transform/MCM and Transform/MIN protocols, and 2.8 and 2.4 for the TransRot/MCM and TransRot/MIN protocols.

The computational time spent by the TransRot/MCM algorithm is split roughly evenly between the initial placement stage and the refinement stage. The Transform/MCN protocol spends the majority of the time in the refinements stage. A combination of the new Transform initial placement algorithm and MIN refinement consistently generating models approximately 5–10 times faster than the previously published docking algorithm.

MIN refinement has more consistent run time when compared to MCM refinement. Each round of repacking during MCM refinement requires that the interactions between atoms in the binding site are recomputed. As the computational complexity of this operation increases with the square of the number of atoms in the protein/ligand interface, the docking of ligands into larger binding pockets takes substantially longer when using MCM refinement which contributes to the observed changes in timing consistency.

### Use of the Transform mover improves sampling efficiency

Using the Transform and TransRot initial placement algorithms 150 initial placement trajectories for each protein in the 43 protein CSAR benchmark set were generated. After each step in the trajectory, the RMSD to the experimental ligand position was computed. S2 Fig illustrates the impact of the Transform algorithm on sampling efficiency. While the TransRot algorithm samples models with RMSD to the experimental structure of less than 2.0 Å only 0.16% of the time, the Transform algorithm samples such models 7.0% of the time.

### The Transform algorithm improves docking success rate

**Fig 4:**
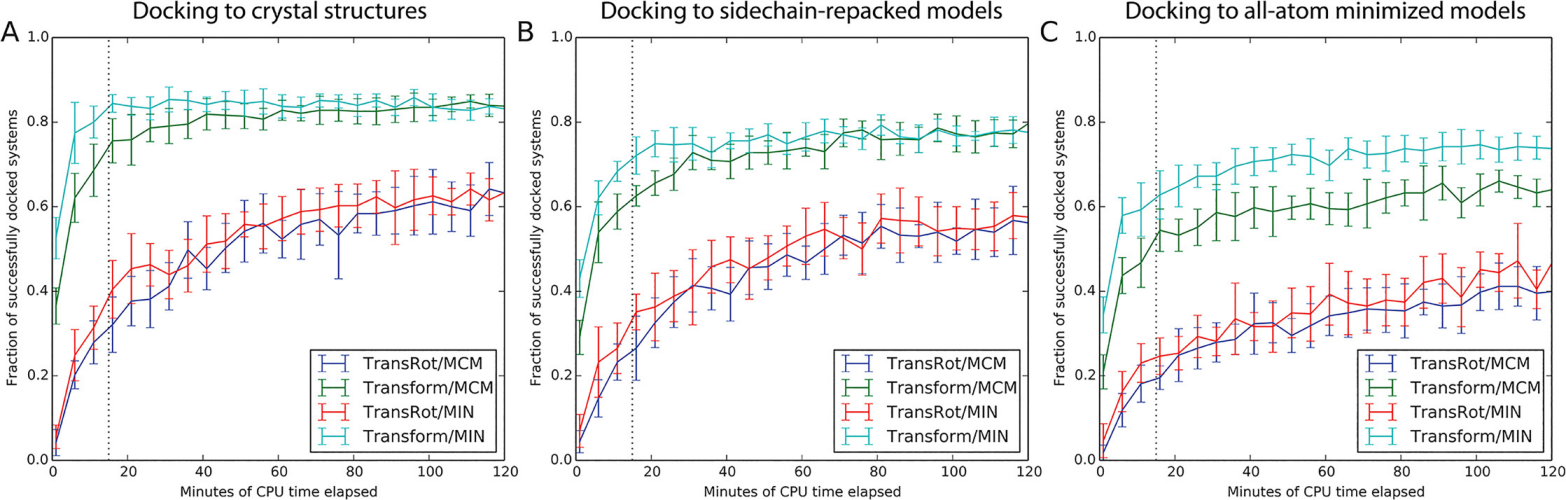
The fraction of protein systems in which the lowest scoring model has an RMSD of less than 2.0 Å to the native structure as a function of CPU time using the four RosettaLigand docking algorithms and three starting protein models. A) Experimental structures, B) models in which only the sidechains are repacked, and C) models in which all atoms have been minimized using the Rosetta energy function. A large pool of models were generated, and random subsamples were taken corresponding to time points at 5 minute intervals. The number of structures included in each time point was based on the average time to generate a model for each algorithm. 20 random samples were taken for each time point, and the means are plotted, with the error bars representing the standard deviation. Docking protocols which make use of the Transform algorithm are reliably converged after approximately 15 minutes (dotted line).

**Fig 5:**
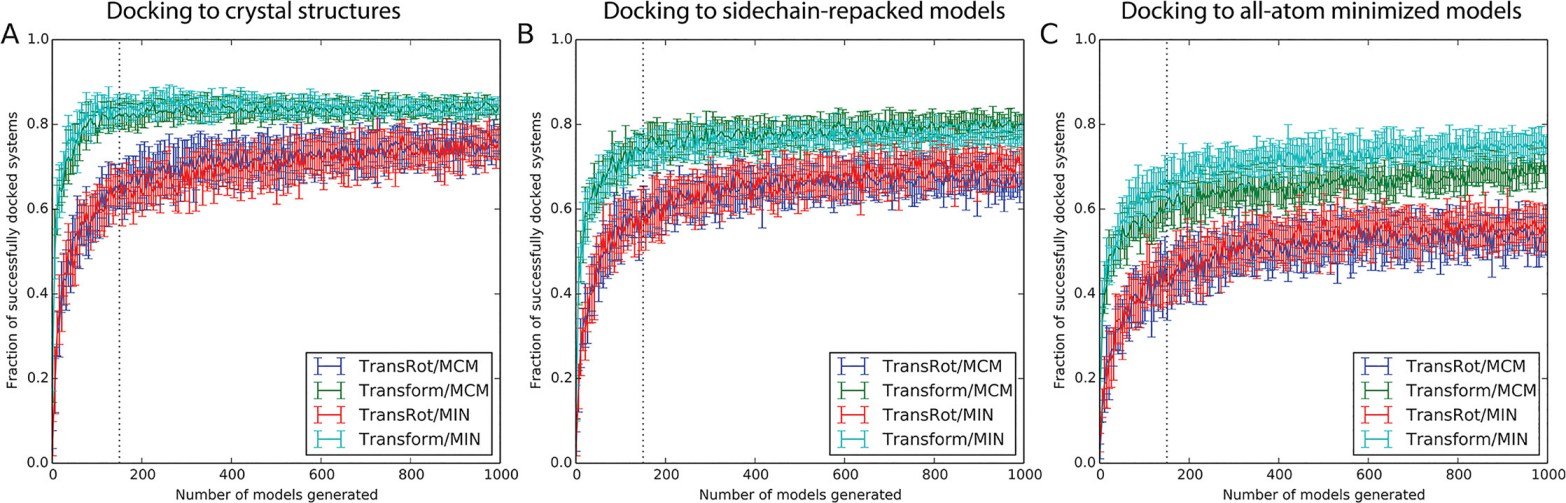
The fraction of protein systems in which the lowest scoring model has a RMSD of less than 2.0 Å to the native structure as function of the total number of structures generated using the four RosettaLigand docking algorithms and three starting protein models. A) Experimental structures, B) models in which only the sidechains are repacked, and C) models in which all atoms have been minimized using the Rosetta energy function. A large pool of models was generated, 20 random subsamples were taken for each point, and the means and standard deviation are plotted. Docking protocols which make use of the Transform algorithm are reliably converged after approximately 150 models (dotted line).

Fig 4 and Fig 5 plot the fraction of protein/ligand complexes for which the lowest scoring binding position is less than 2.0 Å RMSD as a function of total Central Processing Unit (CPU) time and number of models generated, respectively. Thus the choice of the initial placement algorithm is far more important than choice of low resolution scoring method or refinement method. Docking protocols which make use of the Transform initial placement algorithm can reliably dock an additional 10–15% of models within roughly 15 minutes of CPU time, or 150 models, compared to protocols which use the TransRot initial placement algorithm. The choice of refinement algorithm appears to play little role in the overall performance of the docking protocol, except in the case of the previously published protocol (TransRot/MCM), in which case docking performance begins to approach the Transform based protocols after roughly 800–1000 models have been generated (Fig 5). This observed behavior is consistent with previously published studies of RosettaLigand performance using this protocol [13,15,20].

### The new Transform algorithm is still tolerant of backbone and side-chain perturbations while improving success rate

It is clear from Fig 4 and Fig 5 that despite using a pre-computed scoring grid during initial placement, RosettaLigand with the new initial placement algorithm is still tolerant of changes to the side-chain and backbone conformations of the protein binding site. In all tested protocols, the success rate of RosettaLigand decreases as the uncertainty associated with the protein side-chain and backbone atoms increases. In other words, after 1000 models have been generated docking ligands into experimental structures (Fig 5A), The TransRot/MCM protocol has successfully docked 81% of models, while the Transform/MCM protocol has successfully docked 87%. When ligands are docked into relaxed models in which both backbone and side-chain atoms are perturbed (Fig 5C), The TransRot/MCM protocol has successfully docked 60% of models, while the Transform/MCM protocol has successfully docked 75%. This decrease in success rate is expected because the addition of side-chain and backbone perturbation effectively adds noise to the protein structure. However, we see that the Transform/MCM protocol results in a 12% decrease in success rate between relaxed and experimental structures, rather than 21% for the TransRot/MCM protocol. The Transform protocol is more tolerant of inaccurate protein structures than the TransRot protocol. Because Transform algorithm is more likely to place the ligand in a high quality binding position, a greater percentage of total docking time is spent in proximity of the correct binding site and binding position. As a result, the sampling density increases and the overall success rate of RosettaLigand increases relative to the TransRot/MCM algorithm.

As the rotation step of the TransRot initial placement algorithm uses the number of rotatable bonds to determine how many rotations to perform, the amount of time required for the rotation step varies linearly with the number of rotatable bonds. On the other hand, because the Transform initial placement algorithm uses an MCM algorithm with a fixed number of cycles, the time to complete a single model is more consistent compared to protocols that use the TransRot algorithm.

### Details of performance optimization in the Transform algorithm

While the Transform initial placement algorithm performs roughly the same number of sampling moves during initial placement as the TransRot algorithm, the speed improvements seen are a result of differences in how those moves are computed. Rosetta uses a system called the “fold tree” to represent the relationships between rigid body regions of the protein system [15,21]. Since permutations of the protein structure made using the fold tree are performed in internal coordinate space, it is possible to rapidly modify a large system. In the case of ligand docking, however, the system being manipulated is quite small, and the computation of fold tree based permutations quickly becomes dominated by conversions between internal and Cartesian coordinate space. Because only the scoring grids are used for binding position evaluation during the initial placement step, the Transform algorithm can represent the ligand as a list of Cartesian coordinates, which are directly transformed using a rotation and translation matrix. This method of computing ligand permutations is substantially faster than the previous fold tree based method, and accounts for the majority of the observed speed improvement.

### The Transform algorithm improves sampling efficiency and speed

Based on the results of the benchmarking studies described above, the overall effect of the new Transform sampling algorithm is two-fold. First, the quality of binding positions generated during the initial placement stage is improved. Second, the amount of time required to generate the initial placement is reduced. The improvement of the binding positions generated by the initial placement stage results in additional speed improvements by reducing the amount of sampling necessary to produce a high quality binding position. The improved sampling efficiency afforded by the Transform initial placement algorithm both reduces the time that must be spent in high resolution docking, and reduces the total number of models which must be created to reliably produce a correct predicted binding position.

### The majority of performance improvement is driven by the improvements to the initial placement sampling algorithm

**Fig 6:**
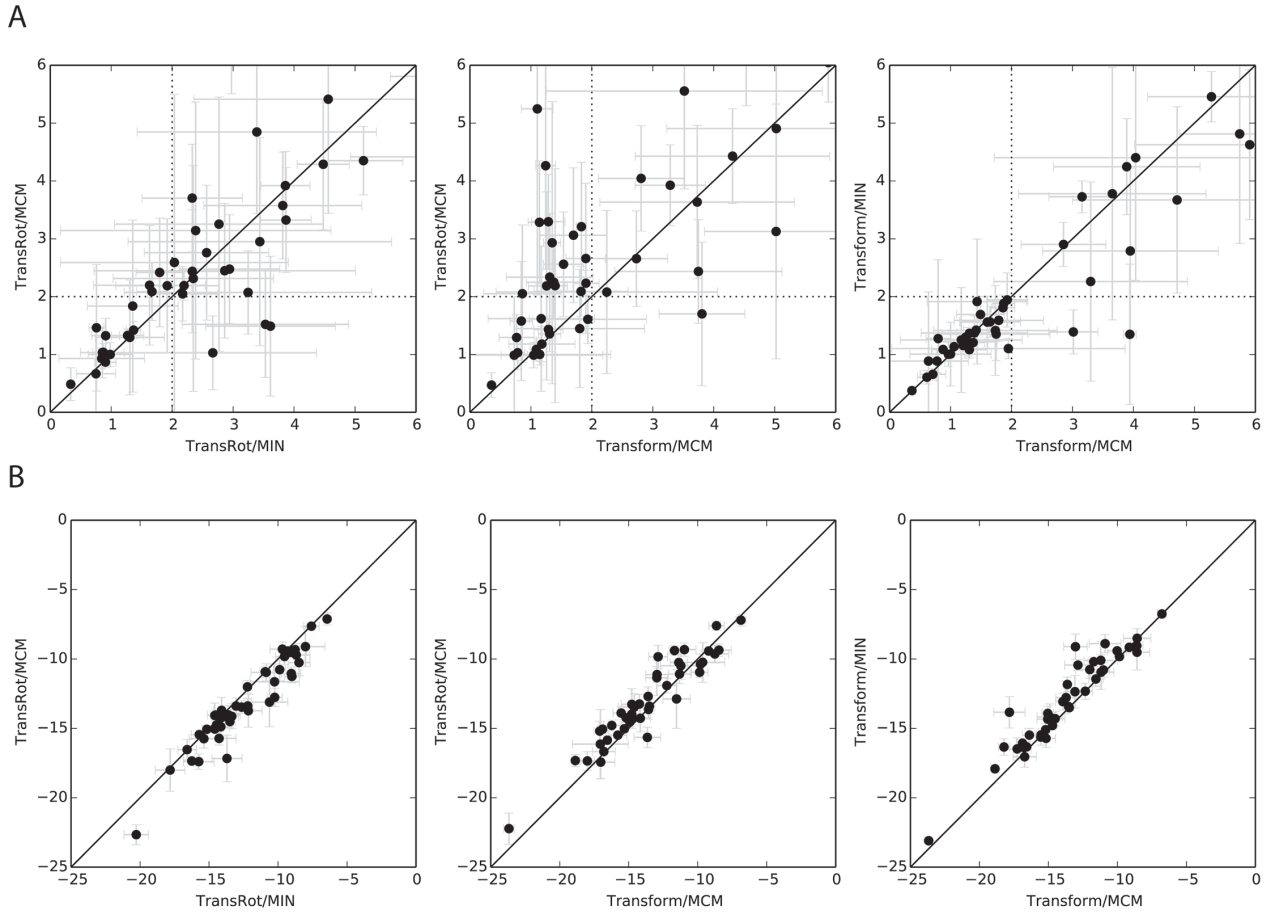
Plots comparing the performance of various docking protocols when docking ligands into relaxed structures. A) For RMSD versus RMSD plots 20 samples of 150 models were collected, and the average of the RMSD of the lowest scoring model is plotted for each protein/ligand system. The standard deviation of these 20 samples is shown with error bars. Dotted lines indicate the 2.0 Å RMSD cutoff used to classify correct vs incorrect binding positions. B) For score versus score plots the change in average all-atom Rosetta score of the lowest scoring model generated by several pairs of docking algorithms.

Fig 6A compares the performance of several of the tested RosettaLigand protocols, and provides further insight into the impact of the various components of the protocol on overall performance. The RMSD versus RMSD plots illustrate specific performance differences comparison between pairs of Rosetta protocols when 1000 models are generated. When the original TransRot initial placement algorithm is used, minimal improvement is observed when the MCM refinement algorithm is used as compared to MIN initial placement (Left). Comparison of the TransRot and Transform initial placement (Center) shows substantial improvement, with 24/43 proteins having improved RMSD, and 15/43 cross the 2.0 Å threshold. Comparison of the MCM and MIN refinement algorithms in combination with the Transform initial placement algorithm shows the two refinement algorithms have nearly identical performance (Right). Improvements in RosettaLigand performance are driven by the new initial placement algorithm.

### Transform initial placement algorithm improves the scores of generated models

Comparison of the scores of the lowest RMSD models generated by protocols using the Transform and TransRot models demonstrate that the use of the Transform initial placement algorithm results in models with slightly lower all-atom scores relative to those generated with the TransRot algorithm (Fig 6B, center) As the energy function is identical between the two protocols, the lower scores indicate that the lower RMSD models generated by the Transform based protocol are also more favorable according to the Rosetta energy function. Because the Transform initial placement algorithm is capable of more efficiently sampling the binding site, it is also more likely to place the ligand in a favorable position prior to refinement and final scoring. Comparing the right most panels of Fig 6A and 6B, we see that while the MCM refinement method results in slightly lower scores than the MIN refinement method, it does not result in an improvement in RMSD. We conclude that several rounds of small ligand perturbations performed by MCM optimize the geometry of the interactions between ligand and protein rather than improving the ligand pose. S2 Table summarizes the data seen in Fig 6.

### Examination of the successes and failures of RosettaLigand illustrates the impact of the Transform algorithm

**Fig 7:**
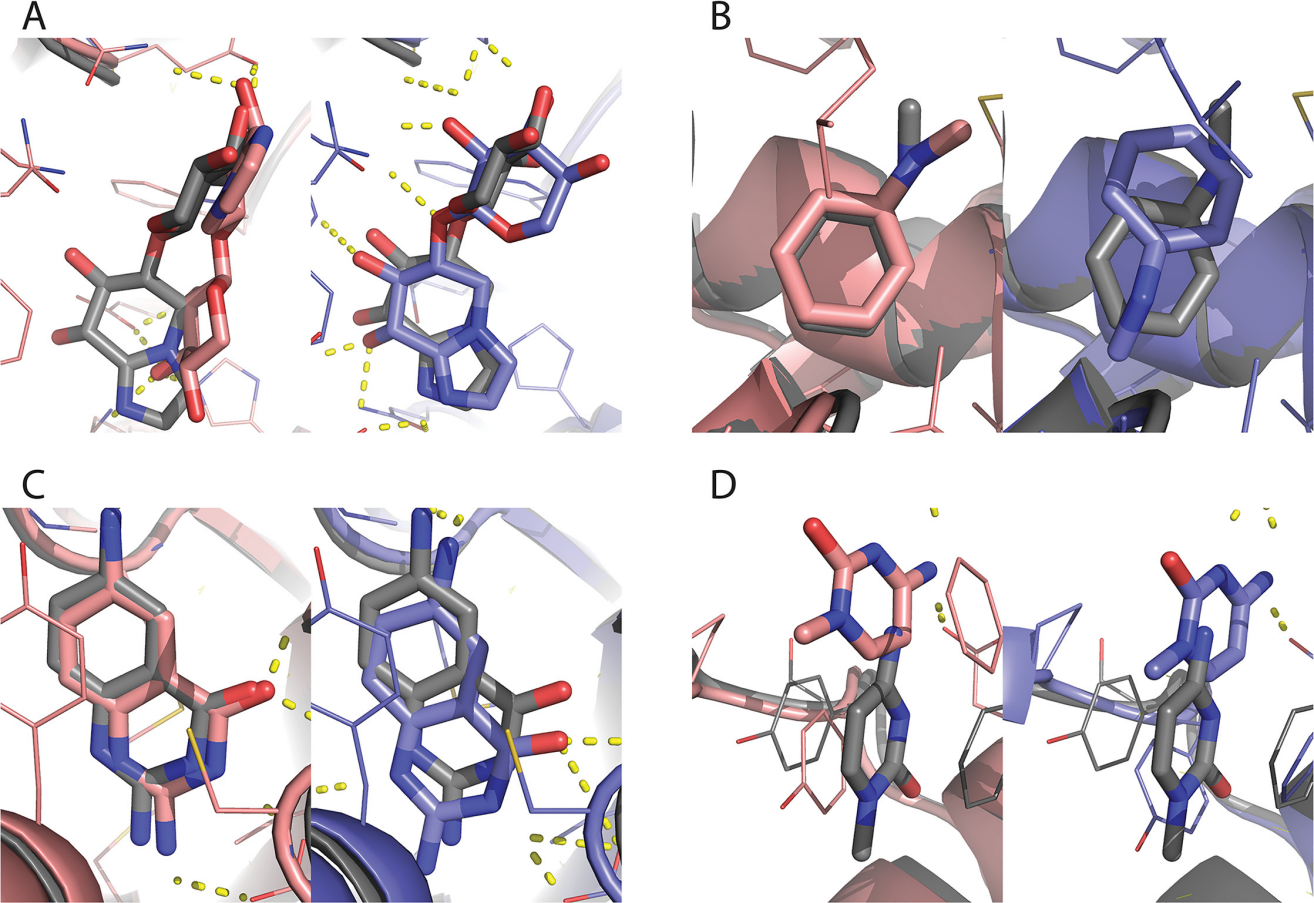
Comparison of specific successes and failures between the RosettaLigand protocols. Native structures are in grey, lowest scoring models generated by the Transform/MCM protocol in blue, and lowest scoring models generated by TransRot/MCM in pink. A) A case in which the TransRot/MCM protocol was unsuccessful but the Transform/MCM protocol was successful (PDB ID: 1fhd). B) A case in which the Transform/MCM protocol was unsuccessful but the TransRot/MCM protocol was successful (PDB ID: 2otz). C) A case in which both methods were successful (PDB ID: 1bky). D) A case in which neither method was successful (PDB ID: 1q4w).

Fig 7 illustrates several examples of the successes and failures of RosettaLigand. Fig 7A illustrates a case in which the Transform/MCM protocol successfully docks a ligand that the TransRot/MCM algorithm cannot dock. The ligand is often flexible and is capable of engaging in hydrogen bonding interactions at multiple sites. As a result, there are likely multiple possible binding positions with relatively low Rosetta energy scores. The more efficient sampling afforded by the Transform initial placement algorithm will increase the probability of sampling the lowest energy, native-like binding pose. In certain cases, the TransRot initial placement algorithm results in improved results over the Transform algorithm. Fig 7B is one such case. In this case, the ligand is extremely small, and can, as such, be placed in a number of similar positions with varying RMSDs. We have seen from previous studies [13] that it is difficult for the Rosetta energy function to distinguish between accurate and inaccurate binding positions of very small ligands. Fig 7C is a case in which both protocols were successfully able to dock the ligand. This case represents a “best case scenario” from the point of view of the Rosetta energy function and sampling algorithm. The ligand is asymmetric and planar, with no rotatable bonds, and the ligand binding site is compact and deeply buried. As a result of this, sampling space is constrained such that the additional initial placement sampling afforded by the Transform mover is unnecessary.

Conversely, Fig 7D is close to a worst case scenario. Here, a very small ligand is bound to a shallow pocket near the surface of the protein. Inspection of the experimental structure shows that the ligand is involved in a π-stacking interaction with two phenylalanine protein residues. This interaction is likely responsible for a substantial part of the total binding energy, but π-stacking interactions are not directly modeled by the Rosetta energy function and as a result are often not be correctly recovered during either initial placement or refinement.

### Despite improved sampling efficiency, K_d_ prediction is difficult

**Fig 8:**
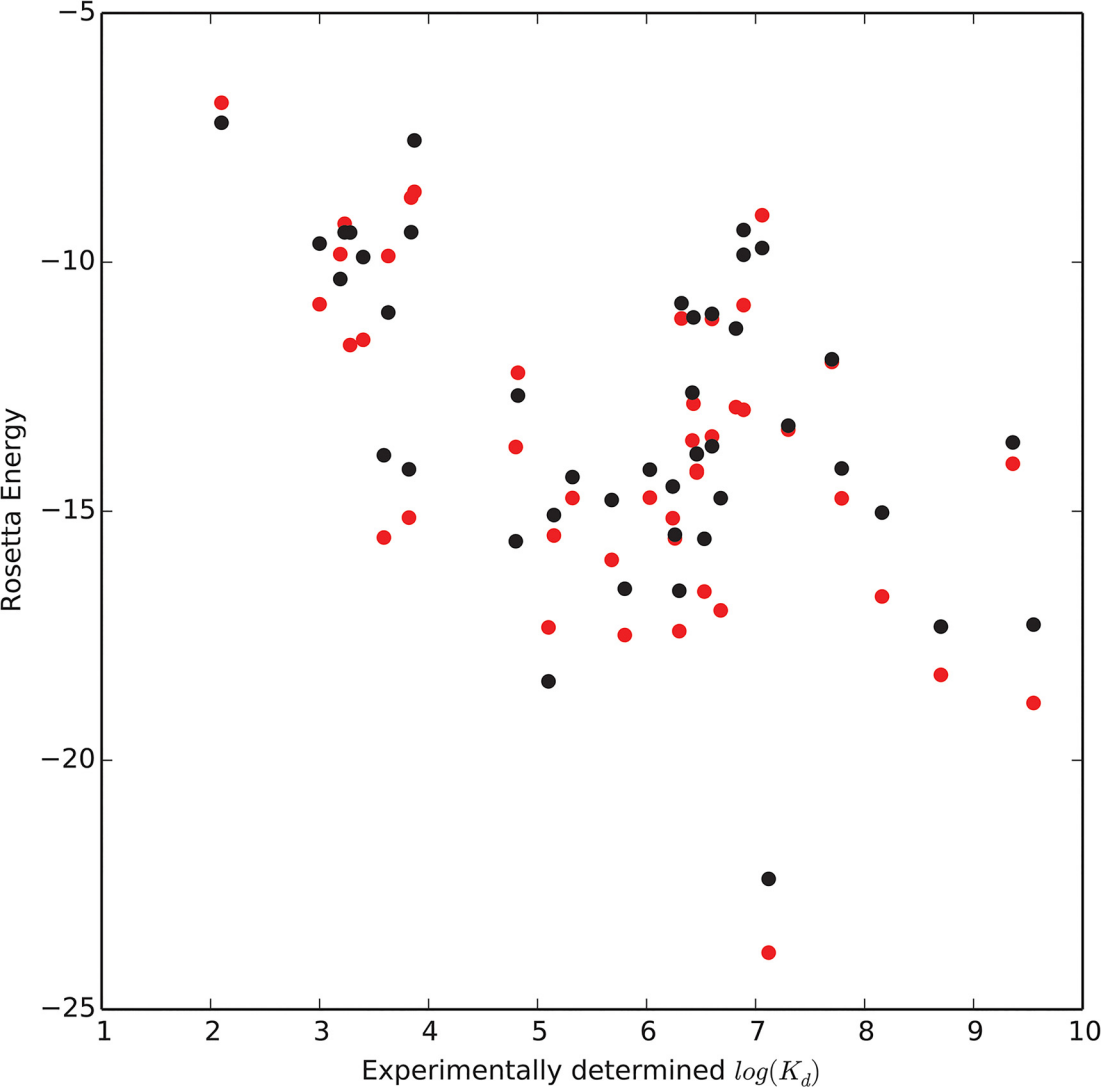
Scatter plots showing the weak correlation between experimental-log(K_d_) and predicted Rosetta energy score for models in the 43 protein benchmark. Scores from models generated using the Transform/MCM protocol are in red while scores from models generated using the Transform/MCM protocol are in black.

While the Transform algorithm results in slightly lower scores, it has no impact on the correlation between Rosetta score and experimentally derived K_d_ values (Fig 8). The correlation coefficient between-log(K_d_) and the Rosetta energy of the models made with the TransRot/MCM protocol is 0.49, while the correlation coefficient for models made with Transform/MCM is protocol improves to 0.54. This observation is in line with previous published studies [14,20] which indicates that the Rosetta energy function, as well as other popular energy functions [11,22] are frequently unable to effectively predict binding affinity.

### Improving the speed of RosettaLigand increases the number of compounds that can be feasibly screened, enabling virtual High-Throughput Screening (vHTS)

While Fig 4 and Fig 5 indicate that both the MIN and MCM refinement algorithms have a similar impact on sampling performance and average run time, the substantially reduced variability in run time of the MIN refinement algorithm illustrated in Fig 3 provides a practical advantage to using MIN rather than MCM for refinement when docking a large number of ligands on a computing cluster, as it allows for more efficient utilization of the available resources of the cluster. For this reason, while the two refinement methods have similar scientific performance, we recommend using MIN refinement, rather than MCM refinement.

Given that RosettaLigand is an “embarrassingly parallel” application, and thus scales linearly with the amount of available CPU resources, a substantial reduction in required run-time per ligand is extremely valuable. By reducing the total processing time per ligand from several hours to approximately 15 minutes, it now becomes possible to screen medium-sized libraries of compounds. This development makes the use of RosettaLigand as a tool for screening ligand libraries computationally feasible for the first time.

### The Transform initial placement algorithm improves the ability of Rosetta to dock challenging protein/ligand complexes

A recurring theme in the development of protein/ligand docking tools is irregular performance of these tools in correctly predicting binding position [9–11,16,23]. While the Transform mover appears to dramatically improve the ability of Rosetta to accurately predict ligand binding poses, some ligands still cannot be correctly docked. The ability to predict a priori whether a ligand can be effectively docked, or at least develop some heuristics to aid in such a prediction, would be valuable. S4 Fig plots the distribution of several ligand descriptors as a function of the ability of Rosetta to successfully dock the ligand. The number of atoms, rotatable bonds, stereo centers, hydrogen bond donors and acceptors are computed, as is the molecular weight, Van Der Waals (VDW) volume and surface area, and girth. Girth is computed as the longest distance between any pair of atoms in the small molecule. All ligand descriptors were computed using the BioChemical Library (BCL). Additionally S5 Fig plots the distribution of several protein/ligand pair descriptors using the native experimental structure. Specifically, the ratio of Rosetta binding energy to Solvent Accessible Surface Area (SASA), the total SASA, the Rosetta Hydrogen bonding energy, the number of residues in the complete protein and at the protein ligand interface, and the packing statistic [24]. As before, a ligand is considered successfully docked if the lowest scoring model is within 2.0 Å of the experimental structure. S4 Fig and S5 Fig suggest that smaller, less flexible, and more deeply buried ligands are easier for both Transform and TransRot based docking protocols to handle, and that the Transform protocol is able to recover the binding mode in larger, more flexible, less deeply buried ligands that the TransRot protocol is unable to correctly model. Unfortunately, while the ligands that cannot be successfully docked by either model tend to be larger and more flexible, we can identify no simple rules for predicting reliably if a ligand will be successfully docked using Rosetta, as the distributions of successful and unsuccessful ligands overlap for every descriptor evaluated.

## Conclusion and Future Directions

### The impact of improvements in low resolution sampling

We have shown that improvement in sampling efficiency can have a large impact on ligand docking performance, even in the absence of improvements to the energy function. Despite the relatively small number of degrees of freedom present in a protein/ligand docking simulation compared to other types of protein simulations, the conformational space is sufficiently complex that choosing an optimized sampling strategy is important. We found that the addition of MC sampling to the initial ligand placement caused a dramatic improvement in the quality of ligand binding poses. This confirms that shape complementarity–the property captured by the scoring grids during initial placement–is an important driver of protein/ligand interaction the energy function is flat with respect to the position of individual atoms.

### The scientific relevance of increased algorithm speed

In addition to improvements in scientific performance, the Transform initial placement algorithm also results in a decrease in the total runtime of the ligand docking simulation relative to the TransRot algorithm. This improvement in speed has important scientific implications. First, increased speed increases the number of compounds that can be computationally tested given a fixed amount of CPU resources. As a result of the tremendous size of chemical space [25], the probability of an active compound existing in a randomly selected subset of chemical space is small, vHTS maximizes the library of compounds screened. Transform/MIN takes an average of 9.3 seconds to generate a model, and requires approximately 150 models to reliably generate a high quality binding position, for a total of 1395 seconds per ligand (23.25 minutes). This allows, for example, on a cluster with 32 CPU nodes each running 8 cores to screen 10,000 ligands in approximately 15 hours using the Transform/MIN method, while the TransRot/MCM method would take 534 hours or around 3 weeks.

This speed improvement brings RosettaLigand closer in CPU requirement to alkternative algorithms. While making direct timing comparisons based the published results of docking algorithms can be challenging for the rapidly evolving hardware, FlexX-PHARM took 26.3 seconds per model in 2002 [4], DOCK took 15 seconds per model in 2001 [3], AutoDock Vina LC took 1 minute per model in 2013 [26], and Glide took < 60 seconds per model in 2004 [6].

One advantage of RosettaLigand is the all-atom, fully flexible representation of both protein and ligand during the refinement stage of docking. We conclude that the most likely application of the TRANSFORM/MIN protocol described in this manuscript would be the screening of medium-sized compound libraries to prioritize a smaller set of ligands. At this point, the Transform/MCM protocol ca be used to refine the binding poses, taking advantage of the optimized geometries and energies obtained through the use of the slower MCM refinement.

## Methods

Complete command lines and instructions for the protein preparation are detailed in the supplementary information (S1 Protocol Capture).

### Description of CSAR experimental structure preparation

The original experimental structures from the CSAR dataset were processed to remove existing water molecules, and hydrogen atoms were added using Rosetta. The side-chains and protein backbone were left at the experimental positions.

### Description of CSAR repacked structure preparation

The experimental structures prepared above were repacked in the absence of the ligand using the Rosetta fixbb application. The backbone was kept fixed, and all side-chain positions were allowed to repack [27].

### Description of CSAR relaxed structure preparation

For each of the experimental structures prepared above, ten relaxed models were produced using the Rosetta relax application. During the Rosetta relax protocol, cyclic repacking of the side-chains and gradient based minimization of the backbone are used to perform MCM of the entire protein structure. In this case, all alpha carbon atoms were restrained to within 0.3 Å of the experimental coordinates, to prevent major conformational shifts. Relaxation was performed in the absence of the ligand.

### Description of ligand conformer generation

Conformers were generated for each ligand using the BCL::ConformerGeneration application(unpublished). BCL::ConformerGeneration uses a stochastic fragment assembly approach to conformer generation, utilizing a database of fragment conformations derived from the Cambridge Structural Database. A maximum of 100 conformers were generated per ligand, though the actual number of generated conformers varies based on the structure of the ligand and the number of rotatable bonds. The generated conformers were used to produce params files and ligand rotamer libraries using the protocol detailed in the supplemental information (S1 Protocol Capture).

## Supporting Information

S1 Fig A schematic showing the architecture of the scoring grid manager. The grid manager takes as input protein and ligand models and computes a score based on these scoring grids. Additionally, the grid manager is responsible for generating and updating the information encoded in the scoring grids.
(PDF)

S2 Fig Smoothed histogram of RMSDs for ligand positions sampled using the TransRot and Transform initial placement algorithms. The X axis plots the RMSD of ligand docking models to the experimental structure. The Y axis represents the percentage of models with a specified RMSD. A vertical dotted line indicates the 2Å success criterion cutoff.
(PDF)

S3 Fig A Welch’s T-Test was conducted comparing the success rates between pairs of protocols across a range of numbers of generated models. To reduce noise, a moving average of the T-Test p-value is plotted for each of the three sets of models (A) Experimental structures, B) Repacked experimental structures, and C) Relaxed experimental structures). The horizontal dotted line indicates the statistical significance threshold of 0.05.
(PDF)

S4 Fig Box and whisker plots showing the distribution of various ligand properties amongst subsets of protein/ligand pairs in the 34 protein binding set. “Both fail” is the set of pairs for which both Transform and TransRot protocols were unable to successfully dock a ligand. “Both succeed” is the set of pairs in which both protocol are successful, and “Transform fix” is the set of pairs for which the TransRot protocol is successful and the Transform protocol is unsuccessful.
(PDF)

S5 Fig Box and whisker plots showing the distribution of various protein properties amongst subsets of protein/ligand pairs in the 34 protein binding set. “Both fail” is the set of pairs for which both Transform and TransRot protocols were unable to successfully dock a ligand. “Both succeed” is the set of pairs in which both protocol are successful, and “Transform fix” is the set of pairs for which the TransRot protocol is successful and the Transform protocol is unsuccessful.
(PDF)

S1 Protocol Capture A document documenting the RosettaLigand software and detailing the steps necessary to reproduce the docking study described in the manuscript. (PDF)

S1 Table CSAR summary data. A table showing the PDB IDs, gene names, protein and ligand information of the proteins in the 43 protein benchmark set derived from CSAR.
(PDF)

S2 Table PDB IDs, RMSDs and Scores for lowest scoring models generated by the Transform/MCM and the TransRot/MCM protocols docking ligands into the set of 43 relaxed protein models. (PDF)
