# Supplementary figures and images for: Fully Flexible Docking of Medium Sized Ligand Libraries with RosettaLigand
Source: PLoS One. 2015 Jul 24;10(7):e0132508. doi: 10.1371/journal.pone.0132508 (PMC4514752; doi:10.1371/journal.pone.0132508)

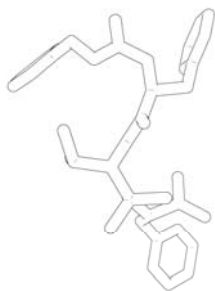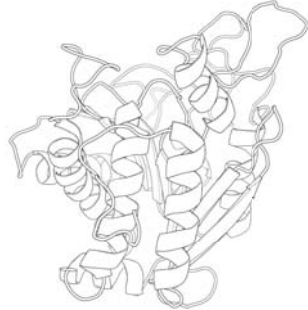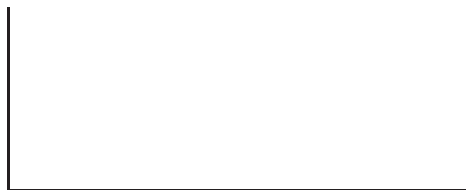

Grid Manager

updates  
score evals

Grid Score

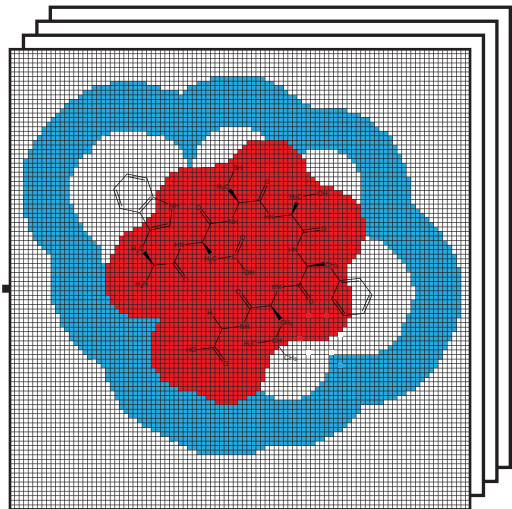

Supplement: S1 Fig — The grid manager takes as input protein and ligand models and computes a score based on these scoring grids. Additionally, the grid manager is responsible for generating and updating the information encoded in the scoring grids. (PDF) [file pone.0132508.s001.pdf]

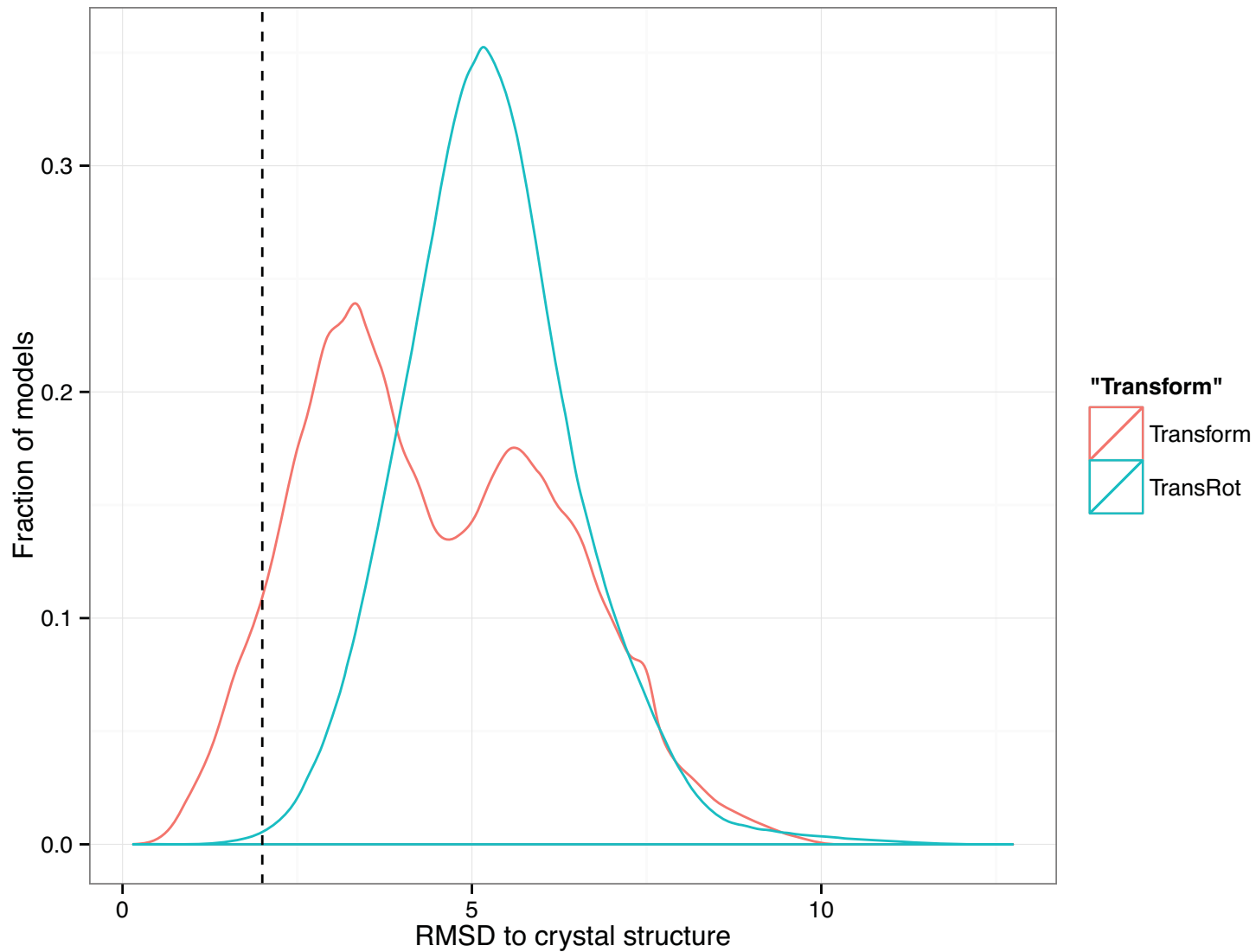

Supplement: S2 Fig — The X axis plots the RMSD of ligand docking models to the experimental structure. The Y axis represents the percentage of models with a specified RMSD. A vertical dotted line indicates the 2Å success criterion cutoff. (PDF) [file pone.0132508.s002.pdf]

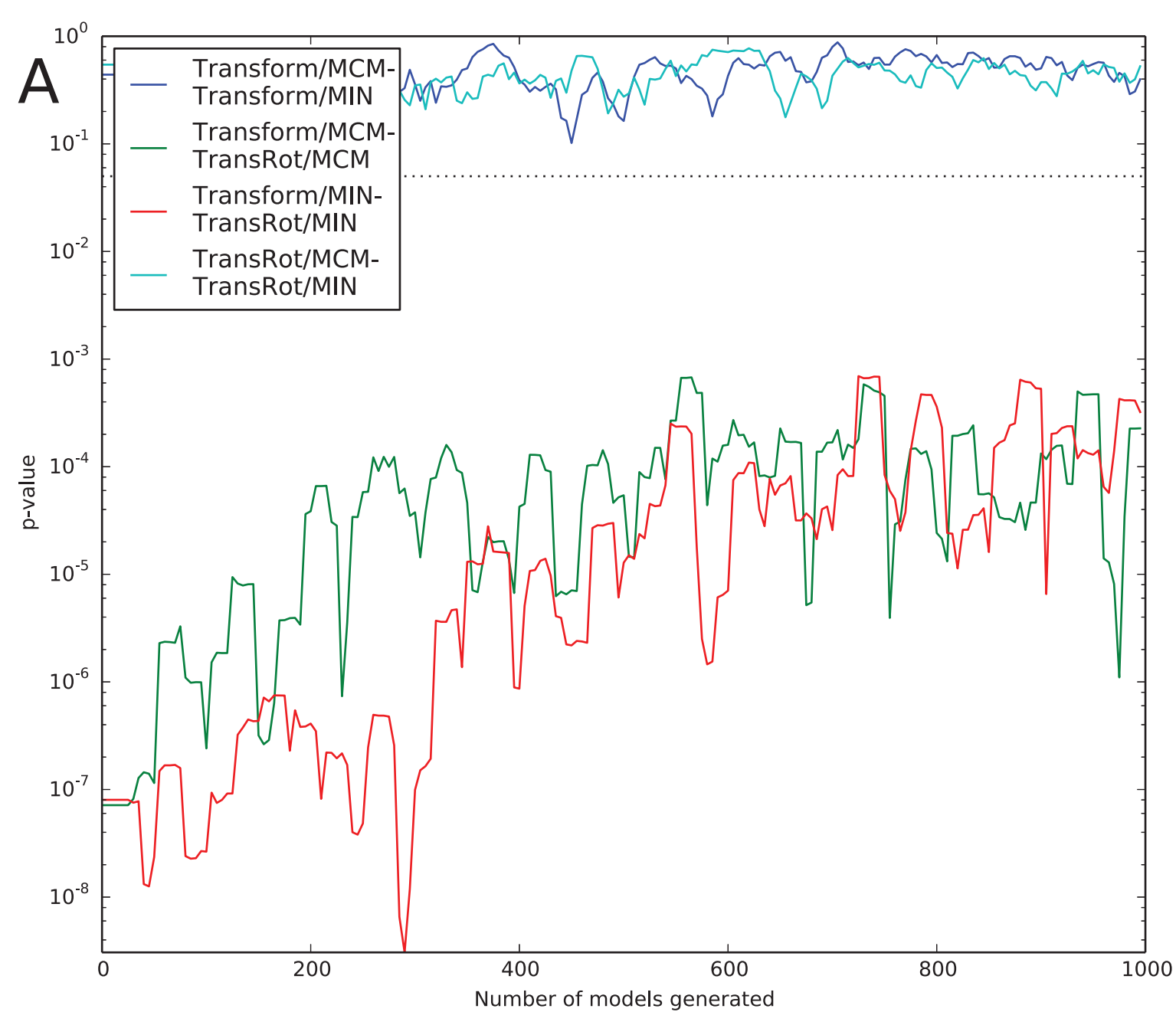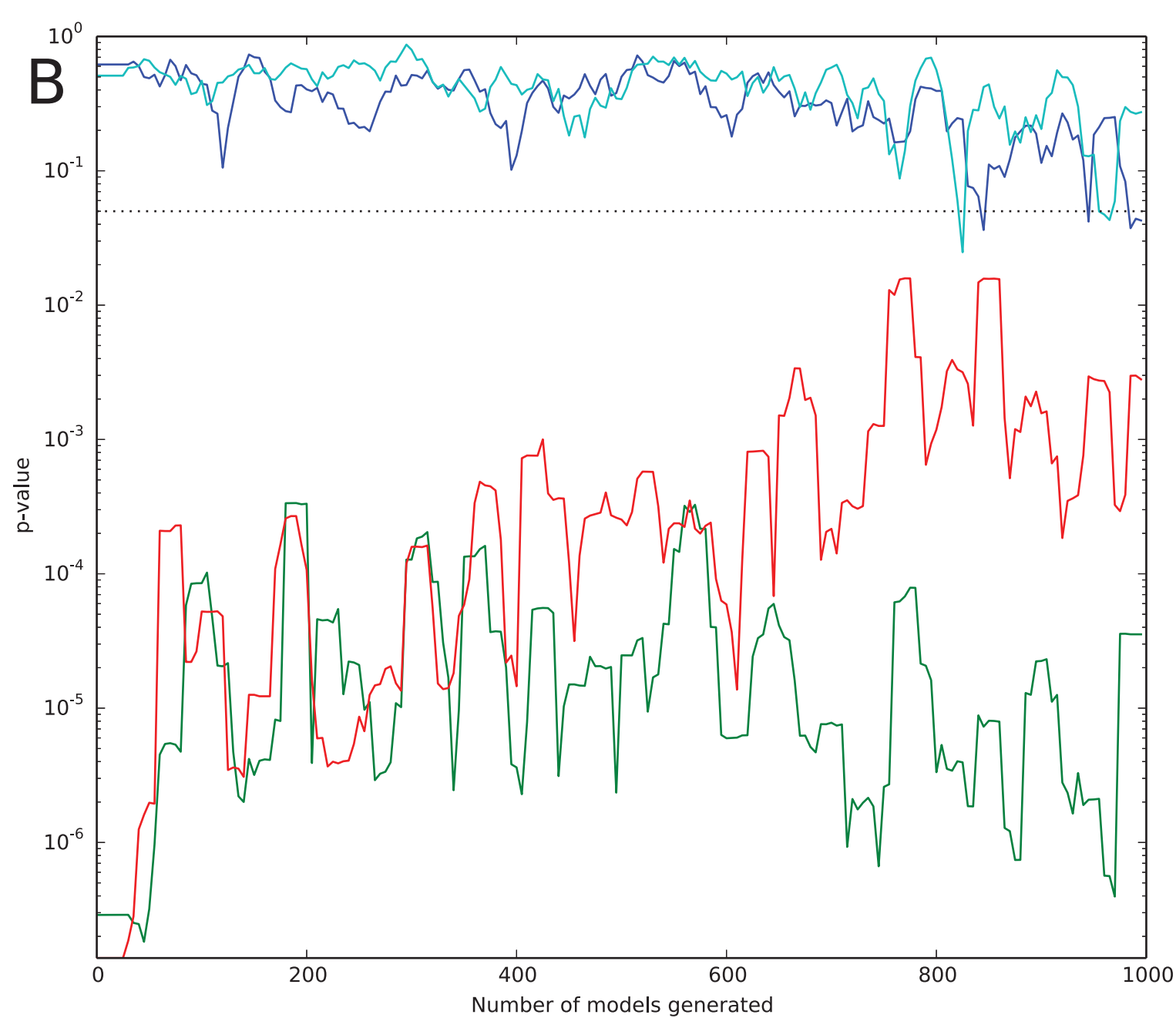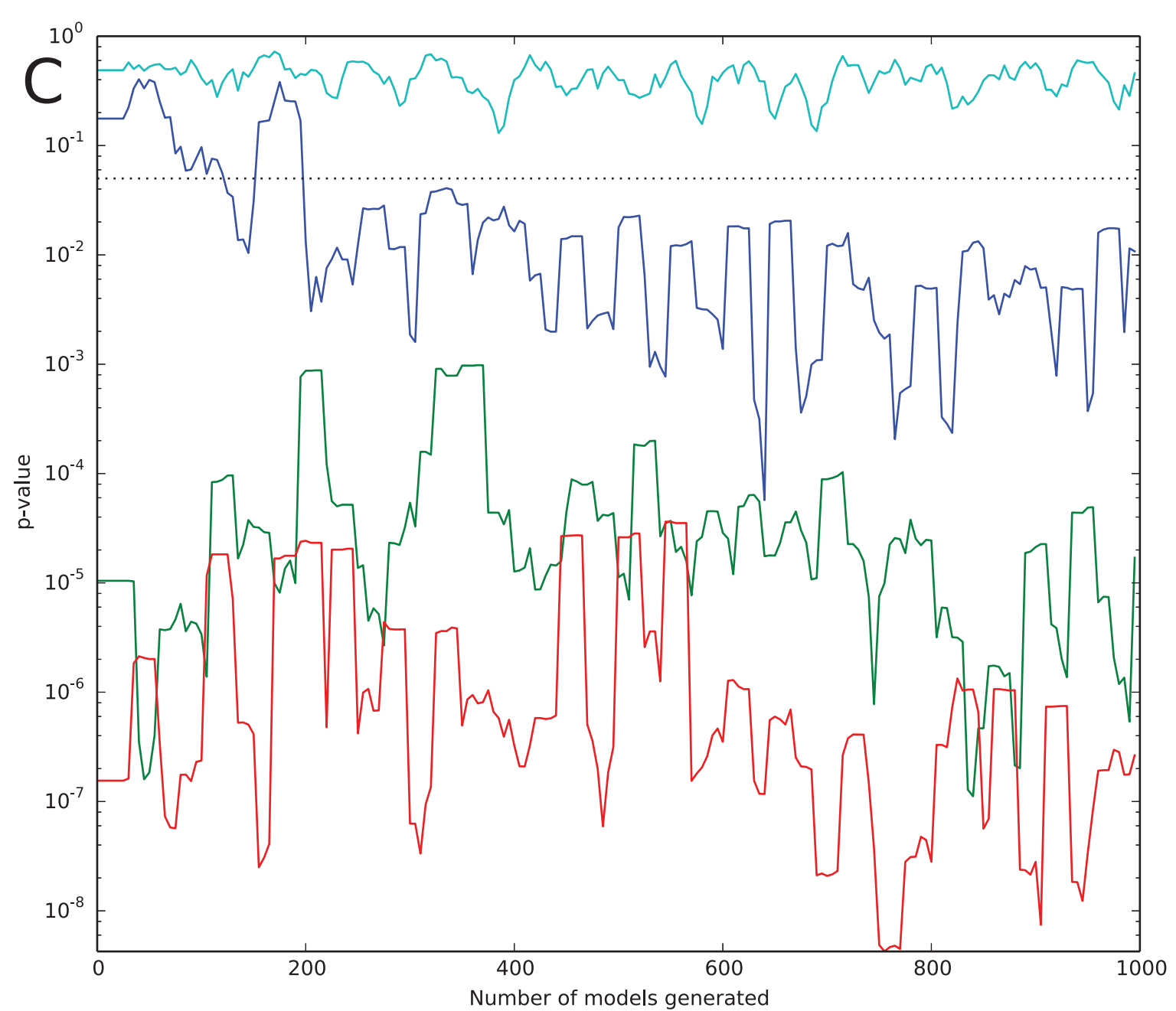

Supplement: S3 Fig — To reduce noise, a moving average of the T-Test p-value is plotted for each of the three sets of models (A) Experimental structures, B) Repacked experimental structures, and C) Relaxed experimental structures). The horizontal dotted line indicates the statistical significance threshold of 0.05. (PDF) [file pone.0132508.s003.pdf]

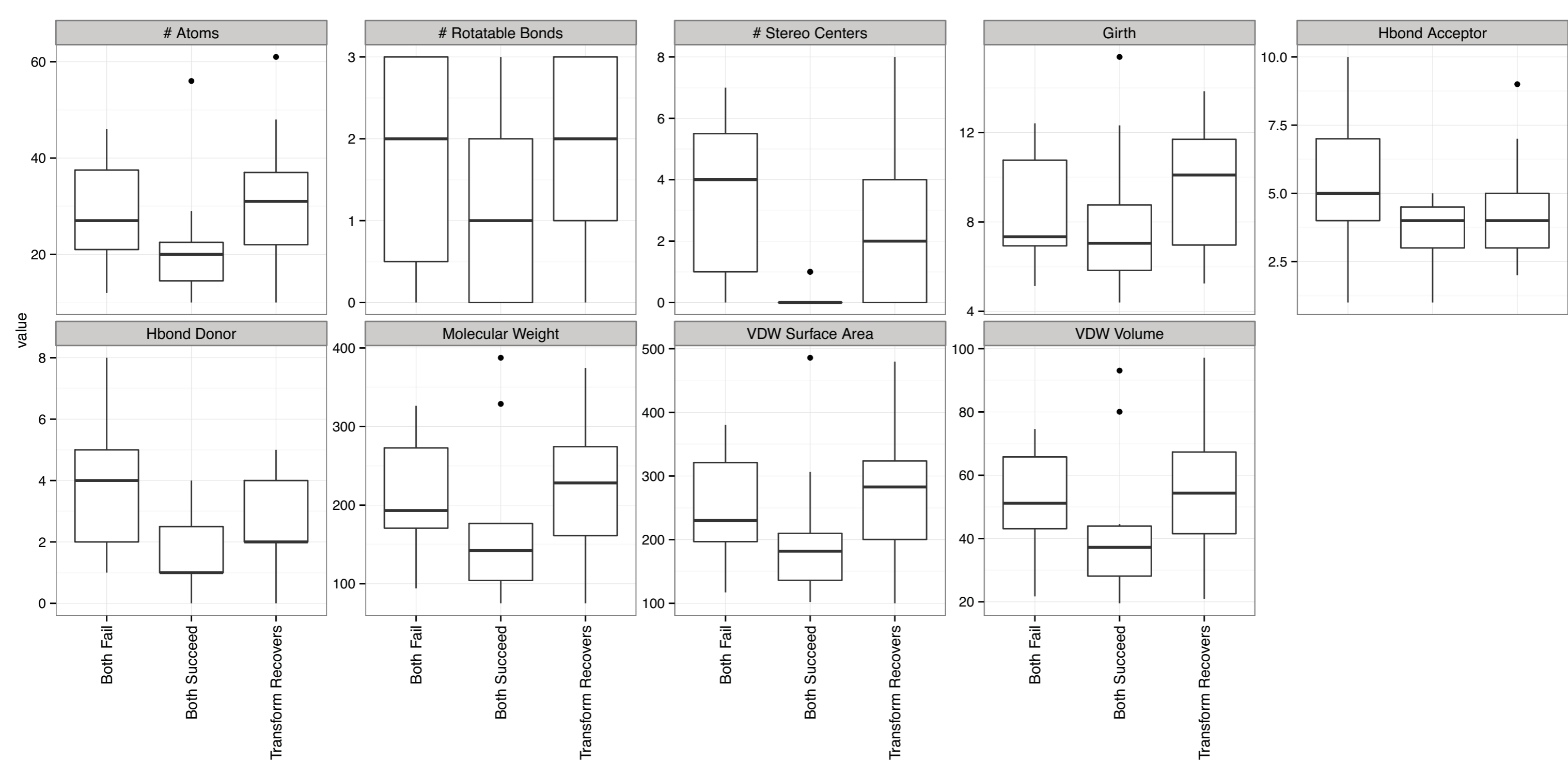

Supplement: S4 Fig — “Both fail” is the set of pairs for which both Transform and TransRot protocols were unable to successfully dock a ligand. “Both succeed” is the set of pairs in which both protocol are successful, and “Transform fix” is the set of pairs for which the TransRot protocol is successful and the Transform protocol is unsuccessful. (PDF) [file pone.0132508.s004.pdf]

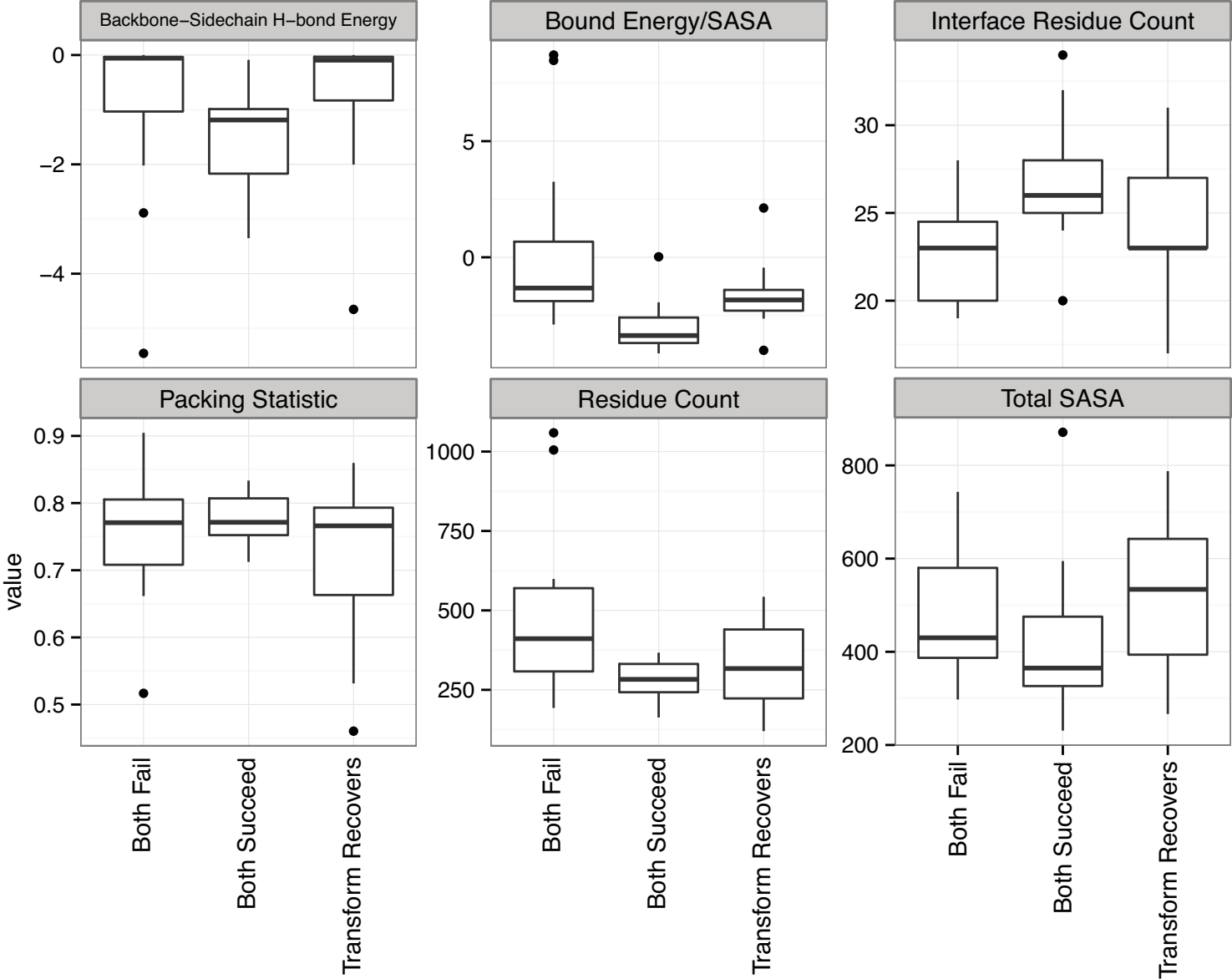

Supplement: S5 Fig — “Both fail” is the set of pairs for which both Transform and TransRot protocols were unable to successfully dock a ligand. “Both succeed” is the set of pairs in which both protocol are successful, and “Transform fix” is the set of pairs for which the TransRot protocol is successful and the Transform protocol is unsuccessful. (PDF) [file pone.0132508.s005.pdf]
