# Supplementary material for: Fully Flexible Docking of Medium Sized Ligand Libraries with RosettaLigand
Source: PLoS One. 2015 Jul 24;10(7):e0132508. doi: 10.1371/journal.pone.0132508 (PMC4514752; doi:10.1371/journal.pone.0132508)
Supplement: S1 Protocol Capture — (PDF) [file pone.0132508.s006.pdf]

Protocol capture

The files associated with this protocol capture can be obtained from www.rosettacommons.org. They are located in the demos directory of the Rosetta distribution in demos/protocol_capture/2015/rosettaligand_transform.

Protocol

Conformer Generation

Conformers can be generated with a number of tools, including MOE and OMEGA. In this case, the Conformer Generation tool included as part of the BCL suite was used. The following command was used to generate conformers:

```
bcl.exe molecule:ConformerGeneration -conformers \
pdb_refinedsupplemented_lib.sdf.bz2 -ensemble \
rosetta_inputs/ligands/all_ligands.sdf    \
-conformation_comparer DihedralBins \
-temperature 1.0 -max_iterations 1000 \
-top_models  100  -bin_size  30.0
-scheduler PThread 8 \
-add_h  -conformers_single_file  conformers
```

You can use any conformer generation tool you have available to you for this step. Your generated conformers should be output to a single Structure Data File (SDF) file. Every conformer must have 3D coordinates and hydrogens added. Conformers of the same ligand should have the same name in the SDF file. For convenience, an example conformer file is provided at `rosetta_inputs/ligands/all_ligands.sdf`.

Params file generation

Params files contain the parameterization information for a ligand. Every ligand or Residue in a protein structure input into Rosetta must have a corresponding params file. Rosetta is distributed with a script called molfile_to_params.py which generates these files. However, this script is generally cumbersome for the generation of more than a small handful of ligands. The protocol below is designed for the preparation of large

numbers of ligands.

All the scripts needed for this process are in the tools directory in the Rosetta distribution. Each of the scripts below would normally be preceded by Rosetta/tools/hts_tools, but this directory prefix has been omitted for brevity.

1. *Split ligand files*

   The conformers for all ligands are initially stored in a single SDF file, but molfile_to_params.py expects one SDF file per ligand. sdf_split_organize.py accomplishes this task. It takes as input a single SDF file, and will split that file into multiple files, each file containing all the conformers for one ligand. Different ligands must have different names in the SDF records, and all conformers for one ligand must have the same name. Output filenames are based on the SHA1 hash of the input filename, and are placed in a directory hashed structure. Thus, a ligand with the name "Written by BCL::WriteToMDL,CHEMBL29197" will be placed in the path /41/412d1d751ff3d83acf0734a2c870faaa77c28c6c.mol. The script will also output a list file in the following format:

```
ligand_id,filename
string,string
ligand_1,path/to/ligand1
ligand_2,path/to/ligand2
```

   The list file is a mapping of protein names to SDF file paths.

   Many filesystems perform poorly if large numbers of files are stored in the same directory. The hashed directory structure is a method for splitting the generated ligand files across 256 roughly evenly sized subdirectories, improving filesystem performance.

   The script is run as follows:

```
sdf_split_organize.py \ rosetta_inputs/ligands/conformers.sdf
\ split_conformers/ ligand_names.csv
```

   Be sure the split_conformers/ directory exists before running the script. Examples of

the output of this script are in `example_outputs/ligand_prep/`

2. *Create Project Database*

   The ligand preparation pipeline uses an SQLite3 database for organization during the pipeline. The database keeps track of ligand metadata and the locations of ligand files. The project database is created using the following command:

```
setup_screening_project.py ligand_names.csv ligand_db.db3
```

   An example of the project database is in example_outputs/ligand_prep

3. *Append binding information to project database*

   The next step is to create a binding data file. The binding data file should be in the following format:

```
ligand_id,tag,value
string,string,float
ligand_1,foo,1.5
ligand_2,bar,-3.7
```

   The columns are defined as follows:

   - **ligand_id** — ligand_id is the name of the ligand, which must match the ligand_id in the file_list.csv file created by sdf_split_organize.py.
   - **tag** — The name of the protein the ligand should be docked into. If a ligand should be docked into multiple proteins, it should have multiple entries in the binding data file. Note that this protocol makes a distinction between protein name, and file name. If you have 4 protein files: foo_0001.pdb, foo_0002.pdb, bar_0001.pdb, and bar_0002.pdb, then you have two proteins with the names foo and bar. The scripts expect that the protein Protein DataBank (PDB) files begin with the protein name.
   - **value** — The activity of the ligand. If you are doing a benchmarking study and know the activity of your ligand, you should enter it here. If you are not doing

a benchmarking study, or if ligand activity is not relevant to your study, value can be set to 1.0 (or anything else). This field is currently only used in a few specific Rosetta protocols that are in the experimental stages, and is typically ignored, so it is safe to set arbitrarily in almost every case.

An example input file is provided. you can insert it into the project database with the following command:

```
add_activity_tags_to_database.py ligand_db.db3\
rosetta_inputs/ligand_activities.csv
```

4. *Generate Params Files*

The next step is to generate params files. make_params.py is a script which wraps around molfile_to_params.py and generates params files in an automated fashion. Params files will be given random names that do not conflict with existing Rosetta residue names (no ligands will be named ALA, for example). This script routinely results in warnings from molfile_to_params.py, these warnings are

not cause for concern. Occasionally, molfile_to_params.py is unable to properly process an SDF file, if this happens, the ligand will be skipped. In order to run make_params.py you need to specify the path to a copy of molfile_to_params.py, as well as the path to the Rosetta database.

make_params.py should be run like this:

```
make_params.py -j 2 --database Rosetta/main/database \
--path_to_params \ Rosetta/main/source/src/python\
/apps/public/molfile_to_params.py \ ligand_db.db3 params/
```

In the command line above, the `-j` option indicates the number of CPU cores which should be used when generating params files. If you are using a multiple core machine, setting `-j` equal to the number of available CPU cores. Be sure that the

params/ directory exists before running the script.

The script will create a directory params/ containing all params files, PDB files and conformer files.

An example of the output params/ directory is found in example_outputs/ligand_prep

5. *Create job files*

Because of the memory usage limitations of Rosetta, it is necessary to split the screen up into multiple jobs. The optimal size of each job will depend on the following factors:

- The amount of memory available per CPU
- The number of CPUs being used
- The number of atoms in each ligand
- The number of conformers of each ligand
- The number of protein residues involved in the binding site.

Because of the number of factors that affect RosettaLigand memory usage, it is usually necessary to determine the optimal job size manually. Jobs should be small enough to fit into available memory.

To make this process easier, the make_evenly_grouped_jobs.py script will attempt to group your protein-ligand docking problem into a set of jobs that are sized as evenly possible. The script is run like this:

```
make_evenly_grouped_jobs.py  --create_native_commands  \
rosetta_inputs/proteins  --n_chunks  1  \
--max_per_job 1000 \
params  rosetta_inputs/proteins  job
```

If the script was run as written above, it would use param files from the directory param_dir/, and structure files from the directory structure_dir/. It would attempt to

split the available protein-ligand docking jobs into 10 evenly grouped job files (-n_chunks). The script will attempt to keep all the docking jobs involving one protein system in one job file. However, if the number of jobs in a group exceeds 1000, the jobs involving that protein system will be split across multiple files (-max_per_job). The script will output the 10 job files with the given prefix, so in the command above, you would get files with names like "output_prefix_01.js". The script will output to the screen the total number of jobs in each file. All the numbers should be relatively similar. If a job file at the beginning of the list is much larger than the others, it is a sign that you should reduce the value passed to -max_per_job. If the sizes of all jobs are larger than you want, increase -n_chunks.

Additionally, the script will take the default ligand positions from the ligand PDB files, and the protein files from the rosetta_inputs/proteins directory, and designate these as the "native" pose of the protein-ligand complex. This feature will allow Rosetta to compute ligand RMSDs automatically, and was used in the benchmarking studies described in the manuscript.

Docking

After following the procedure above to prepare your ligands, you are ready to dock the ligands. The screening job file produced in the previous step contains the paths to the input proteins and ligands and the paths to the necessary params files. In this example, the ligand pdbs are already positioned in the ligand binding site.

RosettaLigand protocols are built in the RosettaScripts framework, a modular architecture for creating RosettaLigand protocols. The rosetta_inputs/xml directory contains all of the rosetta protocols were tested in the manuscript, and any of these XML files can be used with the docking commands described below. See the comments in the XML files for details on the usage and operation of the scripts.

The Rosetta ligand docking command should be run as follows:

```
rosetta_scripts.default.linuxgccrelease \
@rosetta_inputs/flags.txt \
-in:file:screening_job_file rosetta_inputs/job_01.js \
-parser:protocol rosetta_inputs/tr_repack.xml
```

rosetta_inputs/flags.txt contains flags that are always the same regardless of the input file.

This command will dock every protein-ligand binding pair and place the output in the specified silent file. In the benchmarking case described in chapter I, 2000 models were made for each protein-ligand binding pair. However, in a practical application 200 models would be appropriate.

Analysis

Practical analysis

If this protocol is being used for an application project in which the correct ligand binding position is not known, the lowest scoring model for each protein-ligand binding pair should be selected. From that point, we recommend filtering by protein-ligand interface score (interface_delta_X), as well as the packstat score[1] which can be computed through the InterfaceAnalyzer mover. The cutoffs for these filtering steps should depend on the range of scores present, and the number of compounds it is possible to test. After filtering, the selected compounds should be visually inspected. If a crystal structure exists with a known binding pose, the predicted binding poses of the unknown com- pounds should be compared. Additionally, the overall binding poses of the filtered com- pounds should be inspected to assess whether or not they make chemical sense. While this is a qualitative process, human intuition has proven a valuable aid in the drug design process[2].

Benchmarking analysis

Statistical analysis of the benchmarking study provided in this paper was performed using

Python. analysis.ipynb is an iPython Notebook (http://ipython.org/notebook.html) containing the code necessary to reproduce these figures, as well as comments and description of that code. See the iPython documentation for installation and usage instructions.

1. Sheffler W, Baker D (2009) RosettaHoles: rapid assessment of protein core packing for structure prediction, refinement, design, and validation. Protein Sci 18: 229–239. doi:10.1002/pro.8.

2. Voet ARD, Kumar A, Berenger F, Zhang KYJ (2014) Combining in silico and in cerebro approaches for virtual screening and pose prediction in SAMPL4. J Comput Aided Mol Des: 1–11. doi:10.1007/s10822-013-9702-2.
