# Supplementary material for: Fully Flexible Docking of Medium Sized Ligand Libraries with RosettaLigand
Source: PLoS One. 2015 Jul 24;10(7):e0132508. doi: 10.1371/journal.pone.0132508 (PMC4514752; doi:10.1371/journal.pone.0132508)
Supplement: S1 Table — A table showing the PDB IDs, gene names, protein and ligand information of the proteins in the 43 protein benchmark set derived from CSAR. (PDF) [file pone.0132508.s007.pdf]

PDB ID	Ligand ID	Ligand Formula	Protein name	Ligand Name	$log(K_i)$	Weight	AA Count	Citation
2q89	6CS	$C_6H_{10}N_2O_3$	putative abc transporter amino acid-binding protein	(4s,5s)-5-hydroxy-2-methyl-1,4,5,6-tetrahydropyrimidine-4-carboxylic acid	6.3	158.155	257	(Hanekop et al., 2007)
3bgz	VX3	$C_{21}H_{15}NO_2$	proto-oncogene serine/threonine-protein kinase pim-1	2,3-diphenyl-1h-indole-7-carboxylic acid	6.26	313.349	333	(Pierce et al., 2008)
1ukb	BEZ	$C_7H_6O_2$	2-hydroxy-6-oxo-7-methylocta-2,4-dienoate hydrolase	benzoic acid	3.19	122.121	282	(Fushinobu et al., 2005)
1vot	HUP	$C_{15}H_{18}N_2O$	acetylcholinesterase	huperzine a	6.6	242.316	537	(Raves et al., 1997)
2p7g	2OH	$C_{15}H_{16}O_2$	estrogen-related receptor gamma	4,4'-propane-2,2-diyldiphenol	6.53	228.286	251	(Abad et al., 2008)
2rca	GLY	$C_2H_5NO_2$	glutamate [nmda] receptor subunit 3b	Glycine	7.79	75.066	584	(Yao et al., 2008)
2vuk	P83	$C_{16}H_{18}N_2$	cellular tumor antigen p53	1-(9-ethyl-9h-carbazol-3-yl)-n-methylmethanamine	3.82	238.328	438	(Boeckler et al., 2008)
2nta	521	$C_{12}H_9ClN_2O_3S_2$	tyrosine-protein phosphatase non-receptor type 1	5-(4-chloro-5-phenyl-3-thienyl)-1,2,5-thiadiazolidin-3-one 1,1-dioxide	4.8	328.794	299	(Wan et al., 2007)
2are	MAN	$C_6H_{12}O_6$	lectin	alpha-d-mannose	3.28	180.156	504	(Buts et al., 2006)
2z4b	DC8	$C_{18}H_{16}F_2O_3$	estrogen receptor beta	(3as,4r,9br)-2,2-difluoro-4-(4-hydroxyphenyl)-1,2,3,3a,4,9b-hexahydrocyclopenta[c]chromen-8-ol	9.36	318.315	514	(Richardson et al., 2007)
2c94	TSF	$C_{15}H_{23}F_2N_4O_{10}P$	6,7-dimethyl-8-ribityllumazine synthase	3-(1,3,7-trihydro-9-d-ribityl-2,6,8-purinetrione-7-yl) 1,1 difluoropentane-1-phosphate	6.82	488.334	800	(Morgunova et al., 2006)
2b3f	GAL	$C_6H_{12}O_6$	glucose-binding protein	beta-d-galactose	6.03	180.156	2400	(Cuneo et al., 2006)
2bbf	344	$C_9H_7N_5O$	trna guanine transglycosylase	6-amino-3,7-dihydro-imidazo[4,5-g]quinazolin-8-one	5.1	201.185	386	(Stengl et al., 2007)
2pwg	CTS	$C_8H_{15}NO_4$	sucrose isomerase	castanospermine	4.82	189.209	1112	(Ravaud et al., 2007)
2otz	1MR	C_7H_9N	lysozyme	n-methylaniline	3.63	107.153	162	(Mobley et al., 2007)
2ou0	MR3	C_5H_7N	lysozyme	1-methyl-1h-pyrrole	3.23	81.116	162	(Mobley et al., 2007)
2pzv	IPH	C_6H_6O	steroid delta-isomerase	phenol	3.87	94.111	524	(Kraut et al., 2006)
2q88	4CS	$C_6H_{10}N_2O_2$	putative abc transporter amino acid-binding protein	(4s)-2-methyl-1,4,5,6-tetrahydropyrimidine-4-carboxylic acid	5.8	142.156	257	(Hanekop et al., 2007)
1ui0	URA	$C_4H_4N_2O_2$	uracil-dna glycosylase	uracil	7.06	112.087	205	(Hoseki et al., 2003)
1uz1	IFL	$C_6H_{11}NO_4$	beta-glucosidase a	(3s,4r,5r)-3,4-dihydroxy-5-(hydroxymethyl)piperidin-2-one	6.89	161.156	936	(Vincent et al., 2004)
1uz4	IFL	$C_6H_{11}NO_4$	man5a	(3s,4r,5r)-3,4-dihydroxy-5-(hydroxymethyl)piperidin-2-one	3.4	161.156	440	(Vincent et al., 2004)
1v0l	XIF-XYP	$C_{10}H_{21}NO_7$	endo-1,4-beta-xylanase a	piperidine-3,4-diol-beta-d-xylopyranose	6.32	267.276	313	(Gloster et al., 2004)
1ws4	AMG	$C_7H_{14}O_6$	agglutinin alpha chain	alpha-methyl-d-galactoside	3	194.182	612	(Arockia Jeyaprakash et al., 2005)
1y20	1AC	$C_4H_7NO_2$	glutamate [nmda] receptor subunit zeta 1	1-aminocyclopropanecarboxylic acid	5.32	101.104	292	(Inanobe et al., 2005)
2fai	459	$C_{17}H_{24}O_3$	estrogen receptor	4-[(1s,2s,5s,9r)-5-(hydroxymethyl)-8,9-dimethyl-3-oxabicyclo[3.3.1]non-7-en-2-yl]phenol	6.24	276.371	540	(Hsieh et al., 2006)
2fqw	NOS	$C_{10}H_{12}N_4O_5$	membrane lipoprotein tmpc	inosine	6.68	268.226	318	(Deka et al., 2006)
2j78	GOX	$C_6H_{12}N_2O_5$	beta-glucosidase a	(2s,3s,4r,5r)-6-(hydroxyamino)-2-(hydroxymethyl)-2,3,4,5-tetrahydropyridine-3,4,5-triol	6.42	192.17	936	(Gloster et al., 2007)
1bky	1MC	$C_5H_7N_3O$	vp39	1-methylcytosine	3.84	125.129	307	(Hu et al., 1999)
1q4w	DQU	$C_8H_8N_4O$	queuine trna-ribosyltransferase	2,6-diamino-3h-quinazolin-4-one	6.46	176.175	386	(Brenk et al., 2004)
1fcx	184	$C_{26}H_{28}O_3$	retinoic acid receptor gamma-1	6-[hydroxy-(5,5,8,8-tetramethyl-5,6,7,8-tetrahydro-naphtalen-2-yl)-methyl]-naphtalene-2-carboxylic acid	7.12	388.499	235	(Klaholz et al., 2000)
1fh8	XYP-XIF	$C_{10}H_{21}NO_7$	beta-1,4-xylanase	beta-d-xylopyranose-piperidine-3,4-diol	6.89	267.276	312	(Notenboom et al., 2000)
1y93	HAE	$C_2H_5NO_2$	macrophage metalloelastase	acetohydroxamic acid	2.1	75.067	159	(Bertini et al., 2005)
1lhw	ESM	$C_{19}H_{26}O_3$	sex hormone-binding globulin	1,3,5(10)-estratrien-2,3,17-beta-triol 2-methyl ether	8.16	302.408	189	(Avvakumov et al., 2002)
1fh9	XYP-LOX	$C_{10}H_{20}N_2O_9$	beta-1,4-xylanase	beta-d-xylopyranose-3,4,5-trihydoxy-piperidine-2-one-oxime	6.43	312.274	312	(Notenboom et al., 2000)
1s9t	QUS	$C_5H_7N_3O_5$	glutamate receptor, ionotropic kainate 2	(s)-2-amino-3-(3,5-dioxo-[1,2,4]oxadiazolidin-2-yl)-propionic acid	6.6	189.126	518	(Mayer, 2005)
1fhd	XYP-XIM	$C_{12}H_{20}N_2O_8$	beta-1,4-xylanase	beta-d-xylopyranose-5,6,7,8-tetrahydro-imidazo[1,2-a]pyridine-6,7,8-triol	6.82	320.296	312	(Notenboom et al., 2000)
1lnm	DTX	$C_{23}H_{34}O_4$	diga16	digitoxigenin	8.7	374.514	184	(Korndörfer et al., 2003)
1nli	ADE	$C_5H_5N_5$	ribosome-inactivating protein alpha-trichosanthin	adenine	3.59	135.127	248	(Shaw et al., 2003)
1ow4	2AN	$C_{16}H_{13}NO_3S$	pheromone binding protein	8-anilino-1-naphthalene sulfonate	5.68	299.344	258	(Lartigue, 2003)
1r5y	DQU	$C_8H_8N_4O$	queuine trna-ribosyltransferase	2,6-diamino-3h-quinazolin-4-one	6.46	176.175	386	(Lartigue, 2003)
1s38	MAQ	$C_9H_9N_3O$	trna guanine transglycosylase	2-amino-8-methylquinazolin-4(3h)-one	5.15	175.187	386	(Meyer et al., 2004)
1s39	AQO	$C_8H_7N_3O$	trna guanine transglycosylase	2-aminoquinazolin-4(3h)-one	7.7	161.161	386	(Meyer et al., 2004)
1sw1	PBE	$C_7H_{14}NO_2$	osmoprotection protein (prox)	1,1-dimethyl-prolinium	7.3	144.192	550	(Schiefner et al., 2004)

S 2 Table: CSAR summary data. A table showing the PDB IDs, gene names, protein and ligand information of the proteins in the 43 protein benchmark set derived from CSAR.
