# Supplementary material for: Fully Flexible Docking of Medium Sized Ligand Libraries with RosettaLigand
Source: PLoS One. 2015 Jul 24;10(7):e0132508. doi: 10.1371/journal.pone.0132508 (PMC4514752; doi:10.1371/journal.pone.0132508)
Supplement: S2 Table — (PDF) [file pone.0132508.s008.pdf]

| PDB ID | Score of lowest scoring model | | RMSD of lowest scoring model | |
|---|---|---|---|---|
| | TRANSROT/MCM | TRANSFORM/MCM | TRANSROT/MCM | TRANSFORM/MCM |
| 2q89 | -17.1 | -16.1 | 1.0 | 0.8 |
| 3bgz | -15.8 | -15.5 | 6.7 | 4.5 |
| 1ukb | -9.8 | -10.4 | 4.3 | 1.2 |
| 1vot | -13.6 | -13.6 | 4.0 | 2.8 |
| 2p7g | -16.5 | -15.8 | 1.6 | 0.8 |
| 2rca | -14.8 | -14.0 | 1.1 | 1.1 |
| 2vuk | -15.2 | -14.2 | 2.2 | 1.3 |
| 2nta | -13.6 | -15.6 | 1.0 | 1.1 |
| 2are | -11.7 | -9.4 | 2.2 | 1.4 |
| 2z4b | -14.2 | -13.2 | 2.3 | 1.3 |
| 2jj3 | -18.9 | -17.3 | 1.7 | 3.8 |
| 2b3f | -14.8 | -13.3 | 2.7 | 2.7 |
| 2bbf | -17.0 | -17.4 | 1.6 | 1.9 |
| 2pwg | -11.5 | -12.9 | 2.4 | 3.7 |
| 2otz | -9.9 | -11.0 | 1.0 | 1.0 |
| 2ou0 | -9.2 | -9.4 | 2.1 | 0.9 |
| 2pzv | -8.6 | -7.6 | 3.1 | 5.0 |
| 2q88 | -16.8 | -16.7 | 1.4 | 1.3 |
| 1ui0 | -8.8 | -9.6 | 2.1 | 2.2 |
| 1uz1 | -12.9 | -9.8 | 3.3 | 1.3 |
| 1uz4 | -11.4 | -10.3 | 3.2 | 1.8 |
| 1v0l | -11.3 | -11.1 | 2.6 | 1.5 |
| 1ws4 | -9.6 | -10.2 | 3.6 | 3.7 |
| 1y20 | -14.7 | -14.3 | 0.5 | 0.4 |
| 2fai | -14.9 | -14.5 | 2.1 | 1.8 |
| 2fqw | -17.1 | -15.2 | 2.2 | 1.4 |
| 2j78 | -13.6 | -12.7 | 3.9 | 3.3 |
| 1bky | -8.5 | -9.4 | 4.4 | 4.3 |
| 1q4w | -14.6 | -13.5 | 1.6 | 1.2 |
| 1fcx | -23.7 | -22.2 | 1.4 | 1.3 |
| 1fh8 | -11.0 | -9.3 | 2.7 | 1.9 |
| 1y93 | -6.9 | -7.2 | 3.1 | 1.7 |
| 1lhw | -16.9 | -15.1 | 3.3 | 1.1 |
| 1fh9 | -12.9 | -11.1 | 6.0 | 5.9 |
| 1s9t | -11.2 | -10.5 | 4.9 | 5.0 |
| 1fhd | -13.0 | -11.3 | 5.6 | 3.5 |
| 1lnm | -18.0 | -17.3 | 5.2 | 1.1 |
| 1nli | -15.5 | -13.9 | 1.0 | 0.7 |
| 1ow4 | -16.2 | -14.8 | 2.9 | 1.3 |
| 1r5y | -14.2 | -14.3 | 1.2 | 1.2 |
| 1s38 | -15.3 | -15.0 | 1.3 | 0.8 |
| 1s39 | -12.2 | -11.9 | 1.4 | 1.8 |
| 1sw1 | -13.5 | -13.4 | 2.2 | 1.9 |

S 3 Table: PDB IDs, RMSDs and Scores for lowest scoring models generated by the TRANSFORM/MCM and the TRANSROT/MCM protocols docking ligands into the set of 43 relaxed protein models.
